# The bacterial density of clinical rectal swabs is highly variable, correlates with sequencing contamination, and predicts patient risk of extraintestinal infection

**DOI:** 10.1186/s40168-021-01190-y

**Published:** 2022-01-06

**Authors:** Rishi Chanderraj, Christopher A. Brown, Kevin Hinkle, Nicole Falkowski, Robert J. Woods, Robert P. Dickson

**Affiliations:** 1 Division of Infectious Diseases, Department of Internal Medicine, University of Michigan Medical School, Ann Arbor, MI USA; 2 Division of Pulmonary and Critical Care Medicine, Department of Internal Medicine, University of Michigan Medical School, Ann Arbor, MI USA; 3 Institute for Research on Innovation and Science, Institute for Social Research, University of Michigan, Ann Arbor, MI USA; 4 Computational Medicine and Bioinformatics, University of Michigan Medical School, Ann Arbor, MI USA; 5 Department of Microbiology and Immunology, University of Michigan Medical School, Ann Arbor, MI USA; 6 Weil Institute for Critical Care Research & Innovation, MI Ann Arbor, USA; 7 Pulmonary and Critical Care Medicine, University of Michigan Health System, 6220 MSRB III / SPC 5642, 1150 W. Medical Center Dr, Ann Arbor, MI 48109-5642 USA

## Abstract

**Background:**

In ecology, population density is a key feature of community analysis. Yet in studies of the gut microbiome, bacterial density is rarely reported. Studies of hospitalized patients commonly use rectal swabs for microbiome analysis, yet variation in their bacterial density—and the clinical and methodologic significance of this variation—remains undetermined. We used an ultra-sensitive quantification approach—droplet digital PCR (ddPCR)—to quantify bacterial density in rectal swabs from 118 hospitalized patients. We compared bacterial density with bacterial community composition (via 16S rRNA amplicon sequencing) and clinical data to determine if variation in bacterial density has methodological, clinical, and prognostic significance.

**Results:**

Bacterial density in rectal swab specimens was highly variable, spanning five orders of magnitude (1.2 × 10^4^–3.2 × 10^9^ 16S rRNA gene copies/sample). Low bacterial density was strongly correlated with the detection of sequencing contamination (Spearman ρ = − 0.95, *p* < 10^−16^). Low-density rectal swab communities were dominated by peri-rectal skin bacteria and sequencing contaminants (*p* < 0.01), suggesting that some variation in bacterial density is explained by sampling variation. Yet bacterial density was also associated with important clinical exposures, conditions, and outcomes. Bacterial density was lower among patients who had received piperacillin-tazobactam (*p* = 0.017) and increased among patients with multiple medical comorbidities (Charlson score, *p* = 0.0040) and advanced age (*p* = 0.043). Bacterial density at the time of hospital admission was independently associated with subsequent extraintestinal infection (*p* = 0.0028), even when controlled for severity of illness and comorbidities.

**Conclusions:**

The bacterial density of rectal swabs is highly variable, and this variability is of methodological, clinical, and prognostic significance. Microbiome studies using rectal swabs are vulnerable to sequencing contamination and should include appropriate negative sequencing controls. Among hospitalized patients, gut bacterial density is associated with clinical exposures (antibiotics, comorbidities) and independently predicts infection risk. Bacterial density is an important and under-studied feature of gut microbiome community analysis.

Video abstract

**Supplementary Information:**

The online version contains supplementary material available at 10.1186/s40168-021-01190-y.

## Introduction

The past decade has witnessed an explosion in gut microbiome research. Between 2013 and 2017, 12,900 gut microbiome publications were published; prior to that time it took four decades to reach 3000 studies on the topic [1]. This acceleration has been propelled by the advent and widespread use of 16S rRNA gene amplicon-based sequencing [2, 3]. The majority of these gut microbiome studies characterize microbial taxa as relative fractions of a sample sequence library (*relative abundance*). A key limitation of this approach is that it omits the study of the total population density (*absolute abundance*), a fundamental parameter in ecologic analysis [4]. The few studies that have considered the bacterial density of the gut microbiome have found that it is robust against confounding variables [5–7], and correlated with variation in disease status in a manner unappreciated by community composition alone [5, 6, 8].

Rectal swabs are commonly used in gut microbiome studies due to their convenience and ubiquitous clinical use [9–11]. While the bacterial density of *fecal* specimens has been shown to have both methodologic and clinical/prognostic significance [5, 6, 8], the bacterial density of *rectal swabs* has not been reported. Methodologically, rectal swabs may be vulnerable to the sequencing contamination that plagues low-biomass microbiome studies [11–14]. Clinically, it is unknown if rectal swab bacterial density is influenced by clinical exposures (e.g., antibiotic use) or is prognostic of clinical outcomes.

To address these gaps, we quantified bacterial density in rectal swabs from 118 hospitalized patients using droplet digital PCR, an ultra-sensitive quantification technique. We compared bacterial density with community composition (using 16S rRNA gene amplicon sequencing), clinical exposures (e.g., antibiotics and comorbidities), and subsequent risk of culture-confirmed extraintestinal infection.

## Methods

### Study setting and design

We designed a retrospective cohort study using hospital admission rectal swabs previously collected, processed, and analyzed for a study of gut microbiome risk factors for Vancomycin-resistant Enterococcus (VRE) acquisition in 118 patients admitted to the University of Michigan Hospital in 2016–2017 [15]. The infection control practice throughout the study period was to perform routine surveillance for VRE using rectal swabs on eight adult hospital units, including intensive care units, the hematology and oncology ward, and the bone marrow transplant (BMT) ward, in concordance with recommendations from the Centers for Disease Control and Prevention (CDC) the Society for Healthcare Epidemiology of America [16]. All hospitalized patients had routine collection of rectal swabs on admission and weekly thereafter to screen for VRE. Rectal swabs specimens were collected with the BD™ ESwab Regular Collection Kit (Franklin, NJ). The prescribed practice at our institution during the study period was to acquire rectal swabs from patients in left lateral decubitus position. A rectal swab was inserted through the rectal sphincter 2–3 cm, rotated 360°, withdrawn, and checked for the presence of fecal soilage. These swabs were then were stored at − 80 °C.

The current study was a secondary analysis of a previously reported case-control study [15]. In the prior study, cases were defined as subjects with an initial VRE-negative swab followed by a VRE-positive swab when evaluated by selective culture. We matched each case subject to a control subject with an initial VRE-negative swab followed by repeat VRE-negative swab within the same time at risk. An additional matching factor was the unit from which the first positive VRE was recovered for cases or the matched swab after the time at risk for controls. For the current study, we restricted our analysis to admission rectal swabs (one swab per patient).

### Bacterial DNA isolation

After confirming visible fecal soilage of rectal swab specimens, genomic DNA was extracted from rectal swabs, re-suspended in 360 μL ATL buffer (cell lysis solution, Qiagen DNeasy Blood & Tissue kit) and homogenized in fecal DNA bead tubes using a modified protocol previously demonstrated to isolate bacterial DNA [17]. This resulted in a homogenized 500 μL specimen, half of which was used for ddPCR sequencing, and half of which was used for 16S amplicon sequencing. ZymoBIOMICS Microbial Community DNA Standard (Zymo Research) was sequenced as a positive control. Sterile laboratory water and AE buffer (solution of 10 mM Tris-Cl 0.5 mM in EDTA; pH 9.0) used in DNA isolation were collected and analyzed as potential sources of contamination (negative controls). To minimize the potential for batch effects influencing our results, all specimens were extracted by a single laboratory technician using a single extraction kit.

### Bacterial density quantification

Bacterial DNA was quantified using a QX200 Droplet Digital PCR System (BioRad, Hercules, CA). The technique partitions a single sample into 20,000 droplets. A standard PCR reaction then amplifies 16S specific cDNA in each droplet, and each droplet is individually counted by the associated target dependent fluorescence signal as positive or negative. This allows for absolute 16S copy number quantification without generating a standard curve [18–20]. Primers and cycling conditions were performed according to a previously published protocol [20]. To summarize, primers were 5′-GCAGGCCTAACACATGCAAGTC-3′ (63F) and 5′-CTGCTGCCTCCCGTAGGAGT-3′ (355R). The cycling protocol was as follows: 1 cycle at 95 °C for 5 min, 40 cycles at 95 °C for 15 s, and 60 °C for 1 min, 1 cycle at 4 °C for 5 min, 1 cycle at 90 °C for 5 min, all at a ramp rate of 2 °C/s. The BioRad C1000 Touch Thermal Cycler was used for PCR cycling. Droplets were detected using the automated droplet reader (Bio-Rad, catalog no. 1864003), quantified using Quantasoft™ Analysis Pro (version 1.0.596), and imported to R for visualization and statistical analysis. Both sterile water controls, as well as isolation controls, were run alongside rectal swab specimens.

### 16s rRNA gene sequencing

The V4 region of the 16s rRNA gene was amplified using published primers and the dual-indexing sequencing strategy described previously [17]. Sequencing was performed using the Illumina MiSeq platform (San Diego, CA), using a MiSeq Reagent Kit V2 (500 cycles), according to the manufacturer’s instructions with modifications found in the standard operating procedure of the laboratory of Patrick Schloss [17, 21]. All samples were sequenced in a single sequencing run to minimize the potential for batch effects influencing our results.

### Clinical metadata

We collected data from the electronic medical record to describe host health both by the severity of the acute illness that prompted hospitalization and by the severity of chronic disease before hospitalization. We measured acute illness and chronic disease with the Sequential Organ Failure Assessment Score (SOFA score) [22–25] and Charlson comorbidity index [26–28], respectively. We collected data on the antibiotic exposure of patients in the Emergency Department prior to collection of their initial rectal swab. A total 116 of 118 subjects were included in the clinical analysis, as two subjects had sensitive information inaccessible through the medical record.

We used infection-free survival to study the prognostic significance of bacterial density on rectal swabs. We defined extra-intestinal infection as the growth of a bacterial organism by traditional culture media in a site considered by clinicians to be “sterile” (blood, urine, ascites fluid, cerebrospinal fluid, sputum, deep tissue culture) meeting clinical criteria set by major medical societies and the Centers for Disease Control and Prevention [29–35]. Clinical adjudication of positive culture growth led to categorization as colonization, contamination, or clinical infection.

We reviewed the electronic medical record documentation to determine the admitting diagnosis for patients in the cohort. We broadly classified admitting diagnoses into 7 categories: cardiopulmonary disorder (which included congestive heart failure, myocardial infarction, respiratory failure not attributable to pneumonia, and post-operative ICU stay after major cardiac surgery); primary neurologic disorder (which included intracranial hemorrhage, ischemic stroke, or post-operative recovery after major neurosurgery), sepsis syndrome (defined as a presumed infection on admission requiring the use of antibiotics), gastrointestinal disruption (which included inflammatory bowel disease, pancreatitis, bowel obstruction or perforation, or post-operative status after major gastrointestinal surgery), trauma, non-infectious complications of chemotherapy (which included acute renal injury, cytopenia without the presence of neutropenic fever, and nausea and vomiting attributable to chemotherapy), and non-infectious complications of bone-marrow transplantation (which included graft versus host disease as well as nausea and vomiting in the absence of recent chemotherapy administration).

### Statistical analysis of clinical metadata

All analyses were performed using the R statistical programming language (v 4.0.2) [36]. To account for the paired nature of the data, we built a linear mixed-effects model stratified by matched pair status and used clinical covariates to predict log transformed bacterial density. We constructed Kaplan-Meier curves and built a frailty model, also stratified by matched pair status, with the *survival* [37] (v 3.1-8) package in R. Pairwise significance was determined as appropriate by the Wilcoxon test with the Benjamini-Hochberg correction, Tukey’s HSD test, and Mann-Whitney *U* test. All tests used *p* = 0.05 as a threshold for significance.

### 16S gene amplicon analysis

Sequence data were processed and analyzed using the software *mothur* v.1.43.0 [38] according to the standard operating procedure for MiSeq sequence data [17, 39]. We followed the mothur standard operating procedure without deviation, and no low-amplicon sequences were filtered during the analysis. To summarize, the SILVA rRNA database [40] (v. 132, silva.nr_v132.regionV4.align) was used as a reference for sequence alignment and taxonomic classification. K-mer searching with 8-mers was used to assign raw sequences to their closest matching template in the reference database, and pairwise alignment was performed with the Needleman-Wunsch [41] and NAST algorithms [42]. A k-mer-based naive Bayesian classifier [43] was used to assign sequences to their correct taxonomy with a bootstrap confidence score threshold of 80. Pairwise distances between aligned sequences were calculated by the method employed by Sogin et al. where pairwise distance equals mismatches, including indels, divided by sequence length [44]. A distance matrix was passed to the OptiCLUST clustering algorithm [45] to cluster sequences into “operational taxonomic units” (OTUs) by maximizing the Matthews correlation coefficient with a dissimilarity threshold of 3% [46]. OTU numbers were arbitrarily assigned in the binning process and are referred to throughout the manuscript in association with their most specified level of taxonomy (typically genus or family). OTUs were classified using the mothur implementation of the Ribosomal Database Project (RDP) classifier and RDP taxonomy training set 16 (trainset16_022016.rdp.fasta, trainset16_022016.rdp.tax), available on the *mothur* website.

After clustering and classification of sequencing data, we evaluated differences in community structure with permutational multivariate analysis of variance (PERMANOVA) in the *vegan* package (v 2.0-4) [47] in R, and with the *mvabund* [48] package in R. We determined the individual OTU differences driving separation of microbial communities with a random forest classification model built with the *ranger* package (v 0.11.2) [49]. We used the *caret* (v 6.0-84) [50] package in R for cross-validation and hyperparameter optimization. We used latent class regression with the *flexMix* package in R [51] to determine the critical threshold at which rectal swabs are open to sequencing contamination. All OTUs were included in diversity and abundance analyses.

## Results

### The bacterial density of rectal swabs is highly variable and does not correlate with amplicon sequencing depth

**Fig. 1:**
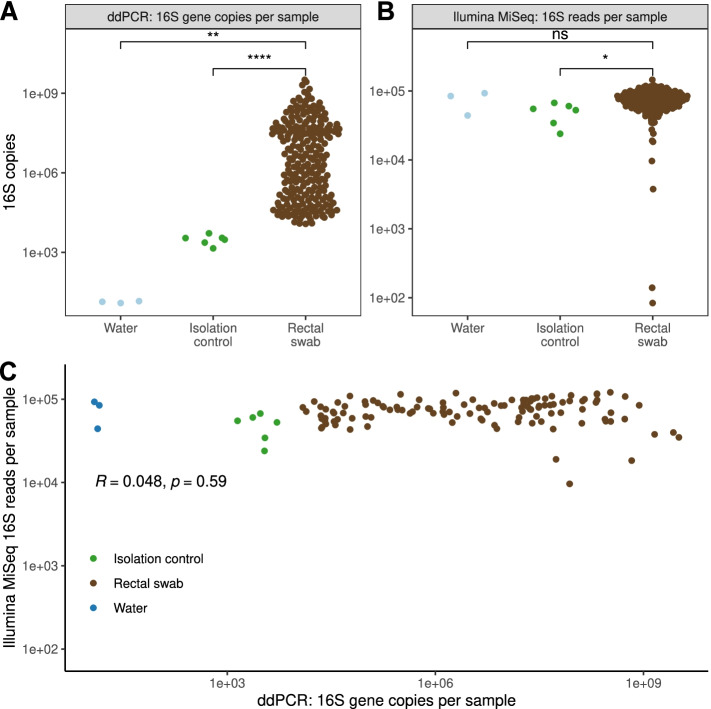
The bacterial density of clinical rectal swabs is highly variable and is not correlated with sequencing depth via 16S rRNA gene amplicon sequencing. We used droplet digital PCR (ddPCR, BioRad) to quantify bacterial density by the absolute copy number of 16S gene in rectal swab specimens from 118 patients admitted to an acute care hospital. We used amplicon sequencing of the 16S rRNA gene (MiSeq, Illumina) to characterize bacterial communities. **A** The bacterial density of rectal swabs was highly variable, spanning 5 orders of magnitude. Rectal swabs specimens had significantly higher bacterial density compared to negative controls (*p* < 0.01 for both comparisons with Tukey’s multiple comparison of means). **B** The number of reads generated via 16S rRNA amplicon sequencing did not distinguish rectal swabs from water control specimens (*p* = 0.99 for rectal swab specimens compared to water controls, *p* = 0.04 for isolation controls, respectively, Tukey’s comparison). **C** The number of amplicon reads per sample was not correlated with the bacterial density of rectal swab specimens (Pearson’s *r* = 0.048, *p* = 0.59). Significance key: ns *p* > 0.05; *p ≤ 0.05; **p ≤ 0.01; ***p ≤ 0.001; ****p ≤ 0.0001

We first sought to establish the variability of bacterial density in rectal swab specimens. Using ddPCR, we quantified bacterial density in 118 rectal swabs from hospitalized patients collected at the time of their admission. We compared this variation with extraction control specimens with sterile water used in DNA extraction (*n* = 3), and isolation control specimens (*n* = 6) (Table 1, Fig. 1). The bacterial density in rectal swab specimens was highly variable, spanning five orders of magnitude (range 1.18 × 10^4^–3.23 × 10^9^ 16S rRNA gene copies/specimen, IQR 4.63*10^7^ 16S rRNA gene copies/swab) (Fig. 1A). All rectal swabs contained greater bacterial density than all negative control specimens: the minimum number of 16S rRNA gene copies/specimen was almost double the maximum number of 16S copies in negative control specimens. We thus concluded that the bacterial density in rectal swab specimens is highly variable, yet consistently greater than that of negative control specimens.

**Table 1:** Summary statistics for droplet digital PCR (ddPCR) and Illumina MiSeq results by specimen type

	Water	Isolation controls	Rectal swabs
**ddPCR 16S copies/sample**			
Mean	14	3.14*10^3^	9.67*10^7^
Median	15	3.22*10^3^	3.17*10^6^
Minimum	9	1.41*10^3^	1.18*10^4^
Maximum	17	5.21*10^3^	3.23*10^9^
Standard deviation	2.4	1.22*10^3^	3.41*10^8^
Interquartile range	3.3	1.17*10^3^	4.63*10^7^
**Illumina MiSeq 16S reads/sample**			
Mean	7.39*10^5^	5.37*10^5^	7.39*10^5^
Median	8.45*10^5^	5.57*10^5^	7.45*10^5^
Minimum	4.42*10^5^	2.39*10^5^	83
Maximum	9.29*10^5^	7.86*10^5^	1.46*10^6^
Standard deviation	2.60*10^5^	1.73*10^5^	2.23*10^5^
Interquartile range	2.44*10^5^	2.09*10^5^	2.95*10^5^

We also observed variation in the number of 16S rRNA gene amplicon reads generated via Illumina MiSeq sequencing (Fig. 1B). We thus asked if the variation in specimen bacterial density (as quantified by ddPCR) correlates with the number of 16S rRNA gene amplicon reads generated via Illumina MiSeq sequencing (as has been assumed in published studies [52, 53]). As shown in Fig. 1 and Table 1, we found far less variation in the number of MiSeq reads than we found in bacterial density. The average number of MiSeq 16S reads was not significantly different between rectal swab specimens and water control specimens (*p* = 0.99; Tukey’s range test) but was significantly different than isolation controls (*p* = 0.04; Tukey’s range test). We next asked if variation in bacterial density correlates with variation in 16S rRNA gene amplicon reads. We found no correlation between the bacterial density of rectal swab specimens and the number of 16S rRNA gene amplicon reads generated via MiSeq sequencing (*p* = 0.59, Fig. 1C). We thus concluded that the number of amplicon reads could not reliably distinguish rectal swab specimens from negative control specimens, and the number of 16S rRNA gene amplicon reads generated via MiSeq sequencing is unrelated to bacterial density, and should not serve as a proxy.

### Rectal swabs are vulnerable to sequencing contamination

**Fig. 2:**
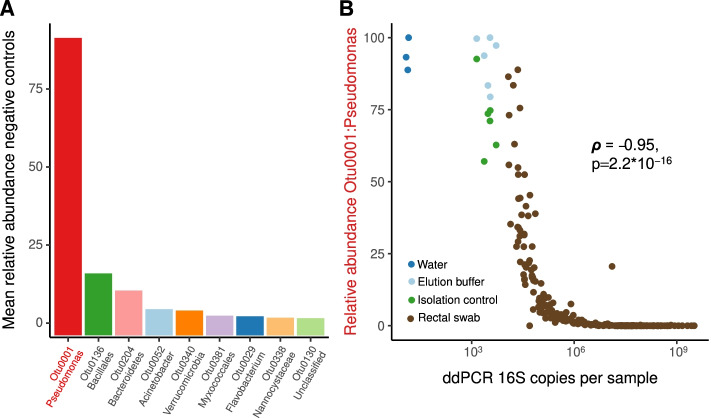
The bacterial density of rectal swab specimens determines their vulnerability to sequencing contamination. **A** The bacterial DNA identified in negative sequencing controls (*n* = 9) was dominated by a single contaminant bacterial taxon (Otu0001: *Pseudomonas*). **B** This same *Pseudomonas* contaminant was present in rectal swab specimens, and variation in its relative abundance was almost entirely explained by the bacterial density of the specimen (Spearman *ρ* = − 0.95, *p* < 2.2*10^−16^)

Low-biomass microbiome studies are vulnerable to contamination due to bacterial DNA present in reagents used in DNA extraction and library preparation [12, 13]. Given the wide variation in bacterial density, we observed in rectal swab specimens, we asked if bacterial communities detected in rectal swab specimens contained any evidence of sequencing contamination. We characterized bacterial communities in rectal swabs and negative controls using 16S rRNA gene amplicon sequencing. We detected unambiguous evidence of sequencing (background) contamination, as negative control communities were dominated by a single bacterial taxonomic group (OTU0001) classified as *Pseudomonas* (Fig. 2A). This same *Pseudomonas* (OTU0001) was detected in rectal swab specimens, and its relative abundance was strongly and negatively correlated with bacterial density (Fig. 2B). Visualizing the relationship between bacterial density and the relative abundance of the *Pseudomonas* contaminant revealed that there appeared to be a critical threshold above which bacterial density was not correlated with the abundance of the contaminant taxa. We used latent class regression to determine a critical threshold where bacterial density became strongly correlated with the relative abundance of the Pseudomonas contaminant, and we determined that above a threshold of 10^6^ copies/specimen, the contaminant OTU was nearly undetected. Below 10^6^ copies/specimen, this *Pseudomonas* taxon was the dominant community member. Formal correlation testing revealed that the bacterial density of rectal swab specimens almost entirely explained the variation in the relative abundance of this contaminant bacterial DNA (Spearman ρ = − 0.95, *p* = 2.2*10^−16^). Altogether, this sequencing contaminant was detected in 62% of all rectal swabs, and in 99% of swabs with a density lower than 10^6^ copies/swab. We thus concluded that rectal swab specimens are vulnerable to sequencing contamination, especially specimens with a density less than 10^6^ 16S rRNA gene copies/specimen.

Our group has previously shown the ability to distinguish *Pseudomonas aeruginosa*, a common hospital acquired pathogen, from non-*aeruginosa Pseudomonas* spp. via 16S rRNA gene amplicon sequencing [54]. We therefore sought to determine if this contaminant OTU may represent *Pseudomonas aeruginosa*. To accomplish this, we analyzed our positive control samples from the ZymoBIOMICS Microbial Community DNA Standard, which contains a known 12% relative abundance of *Pseudomonas aeruginosa*. We noted the presence of 2 *Pseudomonas* classified OTUs in the mock community samples, OTU0001 present at an abundance of 0.2% and 0.1% in two of three of the mock community samples, and OTU0028 present at an abundance of 9–10% in all three mock community samples. Given the large difference in abundance of these two different *Pseudomonas* classified OTUs, one approximating the known relative abundance of *Pseudomonas aeruginosa* in the mock community, and one with extremely low abundance, we inferred that OTU0001 was a non-*aeruginosa Pseudomonas*.

**Fig. 3:**
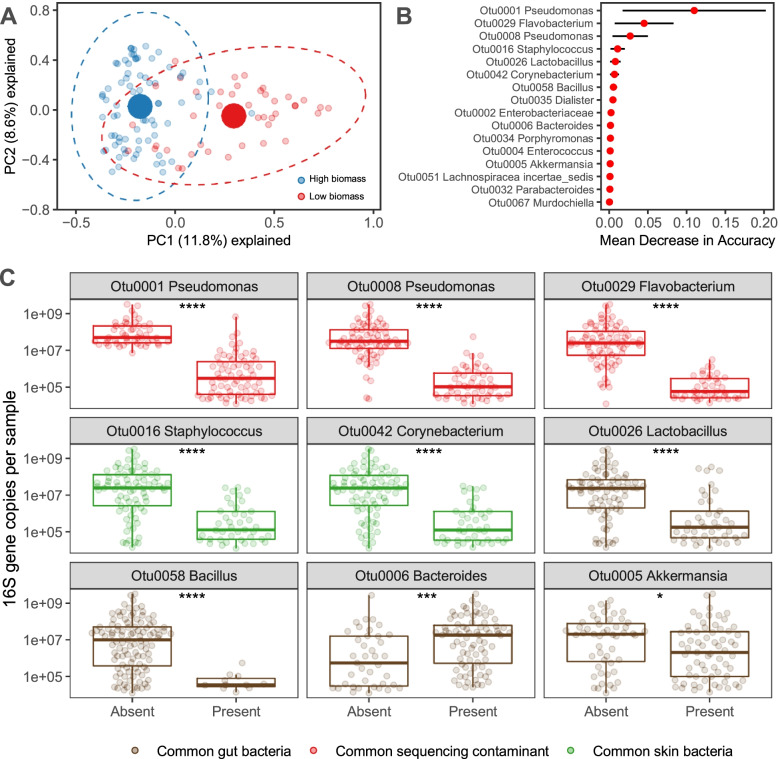
The bacterial DNA identified in low-density rectal swabs is characterized by sequencing contaminants, skin bacteria, and distinct gut bacteria. **A** We visualized the community structure of low density and high density rectal swabs using principal components analysis, which demonstrated a clear separation in community structure. Separation between communities was confirmed as statistically significant with PERMANOVA (*p* < 0.001). **B** A random forest classification model identified the bacterial taxa that drove the differences in community composition across the critical threshold of 10^6^ 16S rRNA gene copies per specimen. **C** After correcting for feature importance bias, 9 bacterial taxa were significantly associated with bacterial density. The presence of common sequencing contaminants (red) and skin bacteria (green) was associated with low bacterial density. Bacteriocin-producing *Lactobacillus* and *Bacillus* spp. were associated with decreased bacterial density, and *Bacteroides* and *Akkermansia* spp. were associated with increased bacterial density. Significance key: ns *p* > 0.05; *p ≤ 0.05; **p ≤ 0.01; ***p ≤ 0.001; ****p ≤ 0.0001

We next investigated whether variation in bacterial density is correlated with variation in community composition. To accomplish this, we interrogated the bacterial community structure of rectal swab specimens and asked how community composition varies with bacterial density. First, we visualized communities using principal component analysis, color-coding specimens by bacterial density (less than or greater than 10^6^ 16S rRNA gene copies/specimen (Fig. 3A). This demonstrated clear separation of specimens by bacterial density, confirmed statistically by PERMANOVA (*p* < 0.001) and by resampling of a generalized linear model (*mvabund*, *p* < 0.001). We next interrogated which specific bacteria drove the overall difference in community composition across specimens varying in bacterial density. To accomplish this, we built a Random Forest classification model and applied a permutation heuristic developed to correct for feature importance bias [55] and identified those features that were significant at *p* < 0.05 (Fig. 3B). The model identified nine bacterial taxa correlated with bacterial density (Fig. 3C). The previously identified *Pseudomonas* contaminant (OTU0001) was the most strongly correlated taxonomic group, followed by two common sequencing contaminants, *Flavobacterium* (OTU0029) and another *Pseudomonas* (OTU0008). These were followed by two commonly reported skin bacteria, *Staphylococcus* (OTU0016) and *Corynebacterium* (OTU0042). Four gut bacterial taxa were correlated with bacterial density, *Bacillus* (OTU000058), *Lactobacillus* (OTU0026), *Bacteroides* (OTU0006), and *Akkermansia* (OTU0005). When we restricted our analysis to swabs above the contamination threshold of 10^6^ 16S copies/sample (above), we identified three significant taxa: the previously identified *Lactobacillus* (OTU0026), as well as *Anaerococcus* (OTU0037) and *Synergistaceae* (OTU 0104). *Lactobacillus* (OTU0026), a known bacteriocin-producing probiotic bacteria [56, 57], was the most strongly correlated taxonomic group in the subset analysis, and was *inversely* correlated with bacterial density (*p* = 9.9*10^−3^). This suggests that the relationship between bacterial density and community composition was not entirely attributable to specimen quality or sequencing contamination, but also reflects authentic correlations across gut communities. We thus concluded that bacterial density and bacterial community composition are correlated, reflecting variation both in sampling/sequencing contamination as well as intrinsic differences within lower gut bacterial communities.

### The bacterial density of rectal swab specimens is correlated with clinical comorbidities and clinical exposures

Having established that bacterial density variation in rectal swab specimens is not entirely attributable to variation in sampling and sequencing contamination, we next interrogated the clinical significance of bacterial density variation. To accomplish this, we compared bacterial density variation with patient clinical characteristics, including demographics, comorbidities, antibiotic exposure, and VRE colonization. Gender, race, specific comorbidities, and reason for admission were not individually associated with bacterial density variation (Table 2, see Supplemental Table 1 for univariate comparisons). Out of 118 patients in the cohort, 116 had data accessible through the electronic medical record and were included in the analysis.

**Table 2:** Demographics and comorbidities of cohort

	*N* = 116 (out of 118 swabs)
**Demographics**	***N*** **(proportion)**
Age (mean ± SE)	60.0±1.37
Female	52 (0.45)
Non-white race	17 (0.15)
**Comorbidities**	
*C. difficile* infection	15 (0.13)
Leukemia	30 (0.26)
Lymphoma	14 (0.12)
Bone marrow transplant	20 (0.17)
Solid organ malignancy	81 (0.70)
Metastatic malignancy	54 (0.47)
Diabetes	47 (0.41)
Coronary artery disease	18 (0.16)
Congestive heart failure	38 (0.33)
COPD	53 (0.46)
Peripheral vascular disease	7 (0.06)
End stage renal disease	46 (0.40)
Connective tissue disease	5 (0.040)
Peptic ulcer disease	16 (0.14)
Cirrhosis	12 (0.10)
Cerebrovascular disease	24 (0.21)
Hemiplegia	10 (0.10)
Dementia	4 (0.03)
Charlson Score (mean ± SE)	4.0 ± 0.19
**Admission diagnosis**	
Cardiopulmonary disorder	22 (0.19)
Gastrointestinal disorder	12 (0.1)
Complications of chemotherapy	36 (0.31)
Neurologic abnormality	3 (0.02)
Sepsis syndrome	34 (0.29)
Non-infectious transplant complication	4 (0.03)
Trauma	5 (0.04)

Recent studies have demonstrated that antibiotics differ in their impact on gut microbiota [58, 59], with piperacillin-tazobactam causing more disruption that other antibiotics [58, 59]. Therefore, we asked if antibiotics differ in their impact on the bacterial density of admission rectal swabs. We first characterized the antibiotic exposure in our cohort (Table 3, Supplemental Table 2). There were a total of 104 antibiotic doses administered to the cohort prior to rectal swab collection. Vancomycin (*n* = 35), metronidazole (*n* = 22), piperacillin-tazobactam (*n* = 20), and cefepime (*n* = 18) were the most administered antibiotics. We compared the mean bacterial density in admission rectal swabs between patients who were and were not exposed to each antibiotic with the Mann-Whitney *U* test. We found that only piperacillin-tazobactam was associated with lower bacterial density (*p* = 0.006), consistent with prior work [58, 59].

**Table 3:** Antibiotic exposure for cohort

16S rRNA gene copies/sample ± SE (log scale)				
	Subjects treated *N* (proportion)	No treatment	Received antibiotic	*P* value
Vancomycin	35 (0.3)	15.18 ± 0.39	14.81 ± 0.53	0.58
Metronidazole	22 (0.19)	15.03 ± 0.37	15.21 ± 0.55	0.79
Piperacillin-tazobactam	20 (0.17)	15.46 ± 0.34	13.15 ± 0.70	**0.006
Cefepime	18 (0.16)	14.95 ± 0.35	15.70 ± 0.72	0.35

**Fig. 4:**
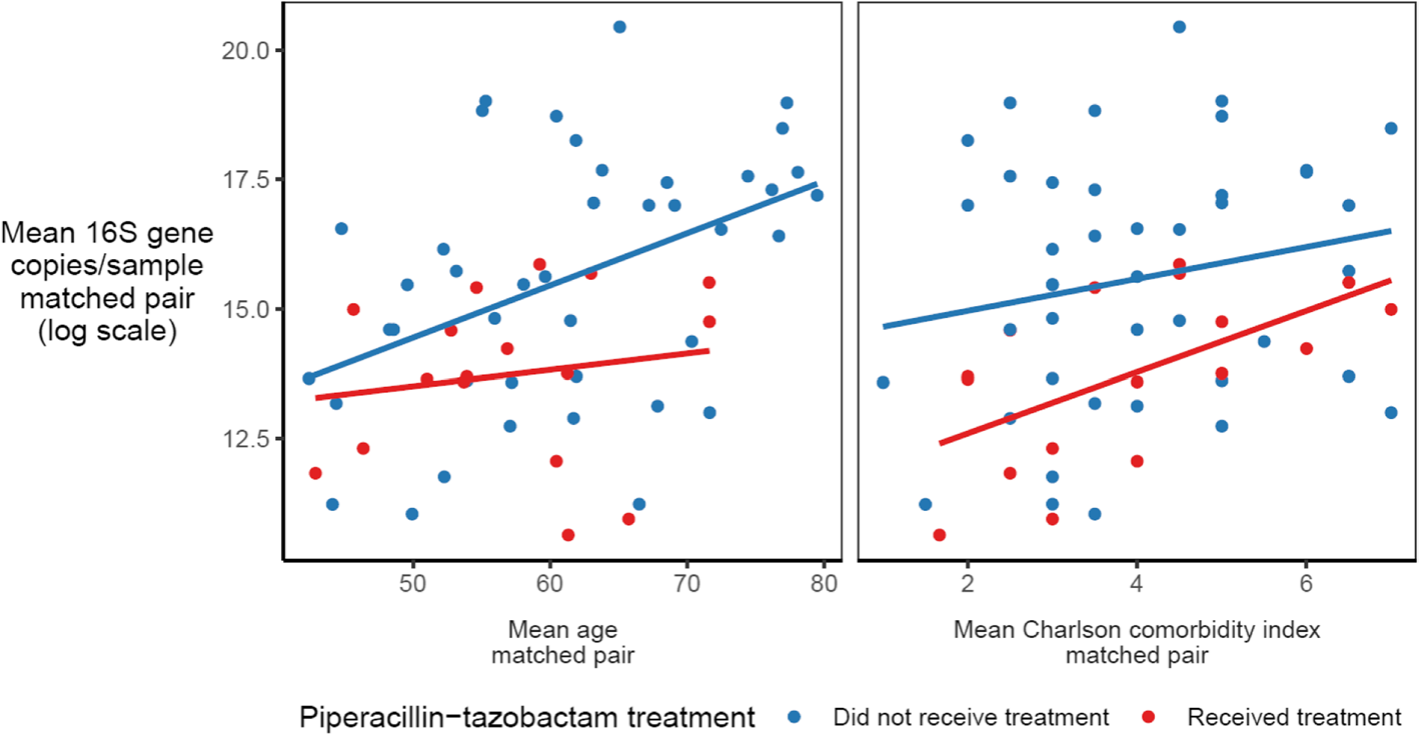
The bacterial density of rectal swabs is strongly associated with piperacillin-tazobactam use. Bacterial density of rectal swabs was compared with clinical features and exposures using multivariable linear mixed-effect regression, stratified by matched case/control pair. Piperacillin-tazobactam exposure was associated with a 1.8 log fold decrease in bacterial density (β = − 1.83, *p* = 0.017). Bacterial density was positively correlated with patient age and medical comorbidities (as described by the Charlson comorbidity score) (increase in decade of age β = 0.43, *p* = 0.03; Charlson comorbidity score β = 0.45, *p* = 0.0040)

Next, we asked if bacterial density was correlated with clinical covariates. To account for the prior matched case-control study design, we built a mixed effects model incorporating the matched pair as a random intercept. We used age, antibiotic exposure, admission diagnosis, chronic comorbidities (via the Charlson comorbidity index [26–28]), and acute severity of illness (via the Sequential Organ Failure Assessment, or SOFA, score [22–25]) to predict the log-transformed bacterial density of rectal swabs. Given our finding that only piperacillin-tazobactam was significantly associated with bacterial burden, it was the only antibiotic included in the model. Increased age and comorbidity burden were all independently associated with increased bacterial density (Table 4, Fig. 4). Every decade of age was associated with a 0.43 ± 0.40 log-fold increase in 16S rRNA gene copies/specimen (*p* = 0.043), and every point increase in the Charlson comorbidity index (signifying more comorbidities) was associated with an average of 0.45 ± 0.29 log-fold increase in 16S rRNA gene copies/specimen (*p* = 0.0040). Piperacillin-tazobactam exposure was associated with a 1.84 ± 1.42 log-fold decrease in 16S rRNA gene copies/specimen (*p* = 0.017). We noted that neither VRE colonization status nor admission diagnosis were associated with bacterial burden after controlling for age, antibiotic exposure, and chronic comorbidities. We concluded that patient demographics, comorbidities, and antibiotic exposure were all associated with variation in rectal swab bacterial density, confirming the clinical and biological significance of this feature of gut microbial communities.

**Table 4:** Fixed effects in linear mixed effects model stratified by matched pair of features associated with bacterial density (log 16S copies/specimen)

	Coefficient (95% CI)	*P* value
Piperacillin-tazobactam	− 1.84 (− 3.26 to − 0.42)	0.017*
Age (decade)	0.43 (0.04–0.83)	0.043*
Charlson comorbidity index	0.45 (0.16–0.74)	0.004*
SOFA Score	0.07 (− 0.13–0.27)	0.51
VRE colonization	− 0.073 (− 1.18–1.06)	0.90
Admission diagnosis		
Cardiopulmonary disorder	0.67 (− 5.421–6.707)	0.84
Neurologic abnormality	2.41 (− 4.534–9.273)	0.52
Trauma	1.35 (− 5.104–7.798)	0.70
Sepsis syndrome	1.45 (− 4.582–7.428)	0.65
Non-infectious transplant complication	− 0.53 (− 7.275–6.276)	0.89
Gastrointestinal abnormality	2.30 (− 3.944–8.433)	0.49
Complications of chemotherapy	0.40 (− 5.607–6.357)	0.90
REML criteria at convergence: 571.6

Having discovered an association between bacterial density and clinical comorbidities and exposures, we next sought to determine if these associations could be due to bias introduced during rectal swab sampling. While we performed an informal quality control check by verifying fecal soilage of the sequenced rectal swabs, in this retrospective study, we were unable to verify that specimen acquisition was performed in a uniform manner for every subject. We thus asked if variation in nursing practices across different patient units was associated with bacterial density. To accomplish this, we compared the bacterial density across unit of admission. We found no collective difference in bacterial density across units (Kruskal-Wallis test, *p* = 0.33, Supplemental Table 3, Supplemental Fig. 1), nor any significant differences between individual units of admission when comparing mean bacterial density with Tukey’s HSD test (Supplemental Table 4). Given the technical difficulty of sampling a mechanically ventilated patient, we next asked if the bacterial density of rectal swabs acquired from mechanically ventilated patients was systematically lower than non-mechanically ventilated patients. We found that the bacterial density of rectal swabs was *increased* among patients receiving mechanical ventilation (Difference in means 1.22 log 16S copies/sample, 95% CI 0.15–2.43 log 16S copies/sample, *p* = 0.043 by *t* test). We added both the unit of admission and mechanical ventilation status to our original model of bacterial density and found that our previous findings still held, and neither of these two possible confounding variables were significantly associated with bacterial density (Supplemental Table 5).

### The bacterial density of rectal swab specimens is associated with subsequent extra-intestinal infections

**Fig. 5:**
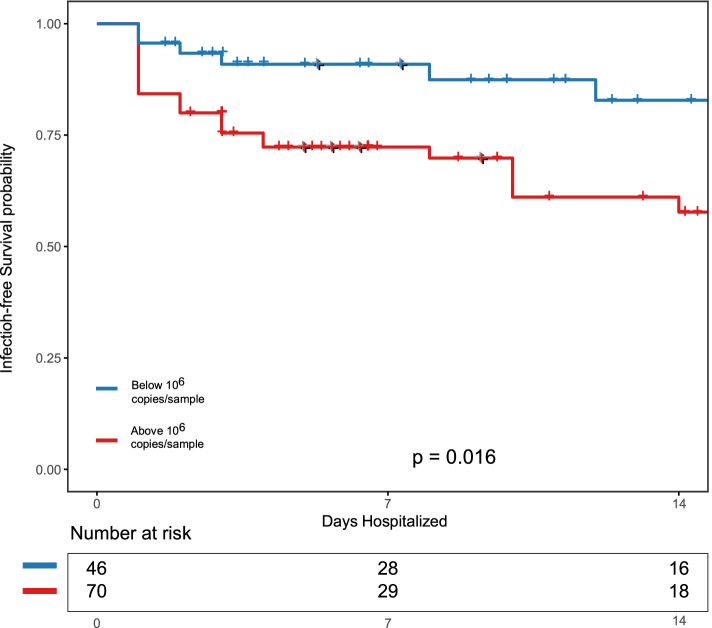
The bacterial density of rectal swabs at the time of hospital admission is predictive of subsequent extra-intestinal infections. Kaplan-Meier curves of infection-free survival in our cohort of hospitalized patients. Cross tick-marks represent censored patients. Using a threshold of 10^6^ 16S rRNA gene copies/specimen, we found that patients with high bacterial density were more likely to have extraintestinal infections at 7 and 14 days following sampling (*p* = 0.016 with stratified log-rank)

Several studies have shown that in hospitalized patients, the gut microbiome serves as a reservoir for potentially infectious pathogens [5, 60–64]. Therefore, we asked if bacterial density variation is associated with subsequent extra-intestinal infections in hospitalized patients (including culture-confirmed bacteremia, pneumonia, urinary tract infections, spontaneous bacterial peritonitis, and soft tissue infections; Supplemental Tables 6 and 7). We first constructed Kaplan-Meier curves of infection-free survival in the cohort. Using a threshold of 10^6^ 16S rRNA gene copies/specimen, we found that patients with low bacterial density were more likely to be alive and infection-free at both 7 and 14 days after sampling (*p* = 0.016 by the log-rank test, Fig. 5). We then constructed a single variable frailty model stratified by matched pairs to predict infection-free survival in the study cohort as a function of the bacterial density at the time of admission. We found that every log-fold increase in bacterial density associated with an increased hazard rate of infection by 17% (*p* = 0.0079).

**Fig. 6:**
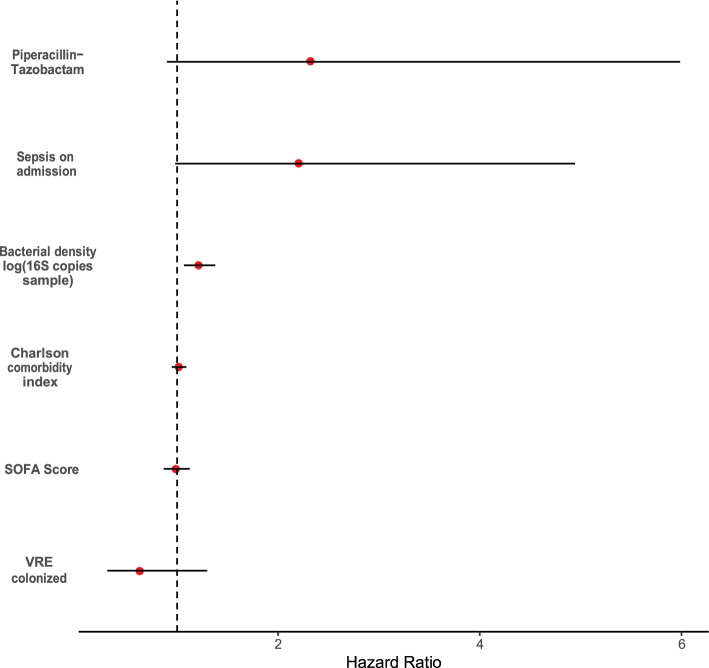
The bacterial density of rectal swabs predicts risk of extraintestinal infection in multivariate analysis. Forest plot for hazard ratio from frailty analysis of infection-free survival, stratified by matched pair. The bacterial density of clinical rectal swabs predicts total infection-free days (*p* = 0.0028) with a hazard ratio of 1.21 for every log fold increase in 16S gene copies/sample. This remained significant after controlling for severity of acute illness, chronic comorbidities, antibiotic use, and admission for sepsis syndrome

Given our findings that age, comorbidities, and antibiotics are associated with bacterial density, we next asked if bacterial density is *independently* associated with subsequent infection, or is merely an indirect measure of susceptibility. We built a multivariable frailty model to stratified by matched pairs to account for the paired nature of the data. We incorporated age, piperacillin-tazobactam exposure, VRE colonization, chronic comorbidities (via the Charlson comorbidity index), and acute severity of illness (via the SOFA score) into the model. We included an admission diagnosis of sepsis syndrome in the model to determine if differences in infection-free survival were driven by new infections or by infections present on admission. In this multivariable model, only bacterial density was associated with subsequent infection (HR 1.21 ± 0.16, *p* = 0.0028, Table 5, Fig. 6). To determine if these associations still held after including possible confounding variables, we constructive an alternative model which included both mechanical ventilation status and unit of admission as covariates. We found that these possible confounding variables were not significantly associated with subsequent infection, and bacterial density remained the only predictor of subsequent infection (Supplemental Table 8).

**Table 5:** Multivariable frailty model of features associated with bacterial infection

	Hazard ratio (95% CI)	*P* value
log (copies 16S/sample)	1.21 (1.067–1.378)	0.003**
SOFA Score	0.99 (0.866–1.125)	0.845
Charlson Comorbidity Index	1.02 (0.947–1.092)	0.650
VRE colonization	0.63 (0.308–1.298)	0.211
Piperacillin-tazobactam	2.32 (0.899–5.986)	0.082
Admission diagnosis of sepsis	2.20 (0.981–4.944)	0.056
Number of events = 37	Likelihood ratio test: *p* <2*10^−8^	Concordance: 0.857

### The bacterial community composition of rectal swabs is associated with subsequent extra-intestinal infection

Having established that bacterial density of rectal swabs is predictive of subsequent infections, we next asked if the observed association was solely an artifact of sampling technique or was reflective of biologically meaningful differences in microbiota structure. To accomplish this, we determined if bacterial community composition could predict extra-intestinal infection. We built a constrained PERMANOVA model stratified by matched case control pairs. We detected a statistically significant separation in the gut communities between patients who did and did not develop extra-intestinal infection (*p* = 0.034). Next, we asked which taxa drove the difference in community structure. We built a random forest classification model incorporating clinical co-variates (the SOFA score and the Charlson comorbidity index), reason for admission, community composition, VRE colonization, and matched pair number to determine which bacterial taxa were predictive of infection. The model identified several taxa predictive of infection after correcting for feature importance bias (Supplemental Table 9). We noted that the same *Lactobacillus* taxa correlated with decreased bacterial density (OTU 0026) was identified as a feature protective against infection (OR 0.47, 95% CI 0.32–0.71, *p* = 0.0002). We noted that the only taxa correlated with both bacterial density and extra-intestinal infection was the previously identified *Lactobacillus* (OTU0026), and no sequencing contaminants were identified as associated with infection (after excluding OTU0001). We thus concluded that both community composition and bacterial density of admission rectal swabs is associated with increased risk extra-intestinal infection.

**Fig. 7:**
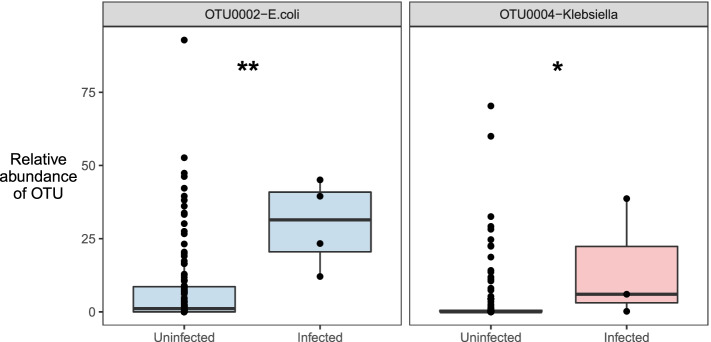
Admission gut microbiota predict enteric gram-negative infection. We found patients with extra-intestinal *E*. *coli* infection had a greater abundance of OTU0002 (*p* = 0.0060 by Wilcoxon-Rank sum test), and patients with extra-intestinal Klebsiella infection had a greater abundance of OTU003 on admission rectal swab (*p* = 0.020 by Wilcoxon-Rank sum test). Significance key: ns *p* > 0.05; *p ≤ 0.05; **p ≤ 0.01; ***p ≤ 0.001; ****p ≤ 0.0001

Prior studies have shown that pathogen colonization at the time of ICU admission is predictive of subsequent infection [64]. We therefore asked if we could detect matches between gut microbiota and distant site clinical isolates. We focused on the most abundant *Enterobacteriaceae* taxa (OTU0002, and OTU0003) and found that patients with extra-intestinal *Escherichia coli* infections had a greater abundance of OTU0002 (*p* = 0.0060 by the Wilcoxon-Rank sum test, Fig. 7), and patients with extra-intestinal *Klebsiella* infection had a greater abundance of OTU003 on admission rectal swab (*p* = 0.020 by the Wilcoxon-Rank sum test). We found that both OTUs were exclusively identical to *Enterobacteriaceae*-classified taxa when comparing closely aligned sequences from the SILVA rRNA database, including *Escherichia coli*, *Enterobacter* spp., and *Klebsiella pneumoniae*. Given the concordance between rectal swab microbiota and distal clinical isolates, we concluded that pathogen colonization detected on rectal swab specimens was predictive of enteric gram-negative infection, consistent with prior studies [64].

## Discussion

Our findings demonstrate that rectal swab bacterial density is highly variable, and that this variability is of methodological, clinical, and prognostic significance. We found that 16S gene amplicon sequencing of rectal swab specimens was vulnerable to sequencing contamination and that the influence of contamination was almost entirely dependent on the bacterial density of the specimen. We found evidence that bacterial density was not merely a marker of sampling adequacy, as it was both associated with clinical comorbidities and exposures and predictive of infection-free survival. Our findings suggest that the bacterial density of rectal swab samples is an essential but overlooked ecologic feature in the study of the gut microbiome in hospitalized patients.

Our key findings are aligned with those of prior studies. In culture-based studies, bacterial density predicted the onset of sepsis and pneumonia among critically ill patients [65, 66], a finding congruent with our culture-independent results. Similar to other studies using culture-independent bacterial quantification, we found that bacterial density had clinical associations unappreciated by standard 16S gene amplicon sequencing [5, 6, 8]. In contrast to prior work, our ultra-sensitive quantification technique (ddPCR) was able to quantify variation in low and high biomass specimens with similar precision [20, 67, 68] and allowed us to quantify the total number of copies of the 16S gene present in a rectal swab specimen without reference to the absolute abundance of individual species. Taken together with prior studies, our findings suggest that the measurement and analysis of bacterial density can provide methodological, clinical, and prognostic insights into gut microbiome studies.

The bacterial density of rectal swabs has methodological importance, as it precisely quantifies the risk of sequencing contamination, an underappreciated challenge in gut microbiome studies. Many of our rectal swab specimens had very low bacterial density, comparable to what is commonly seen in low biomass microbiome studies, such as those of the lung [69, 70]. Given this wide variation, many rectal swabs were vulnerable to sequencing contamination [12–14], a finding compatible with prior studies [11]. To our knowledge, our study is the first to describe the strong association between bacterial density and sequencing contamination, as the level of sequencing contamination in these specimens was negatively correlated with bacterial density. The quantification of bacterial density is thus clarifying in microbiome studies and should be strongly considered as a complementary assay to discriminate legitimate signals from those of background contamination.

We found that bacterial density holds clinical significance, as it was associated with both clinical comorbidities and antibiotic exposure in a manner consistent with prior literature. Previous studies have shown that gut bacterial density increases with increased gut transit time [7]. We found that age and medical comorbidities, two features leading to decreased gut motility and increased transit time [71–73], were positively correlated with bacterial density. Among all administered antibiotics, we found that only piperacillin-tazobactam had a significant effect on the bacterial density of rectal swab specimens. This is concordant with a recent study using 16S amplicon sequencing, which showed that piperacillin-tazobactam caused more disruption to gut microbiota than other antibiotics [58].

Our study found that bacterial density holds prognostic significance, as it predicts the risk of infection during hospitalization**.** When controlling for acuity of illness, chronic medical comorbidities, and age**,** bacterial density remained significant, while these previously validated predictors lost importance. We recently demonstrated that the gut microbiota of hospitalized patients undergoes rapid and profound change [15] and that the majority of bacterial species present on admission are not present later in hospitalization. Given this rapid change, a global metric of bacterial density that captures information about the population rather than individual members of the population may be a more useful index of the dynamic state of the gut microbiome in hospitalized patients. Further study with longitudinal sampling of gut microbiota is needed to investigate this phenomenon.

We acknowledge that some of the observed variation in bacterial density was caused by variation in the specimen acquisition technique, as we were unable to determine whether the clinical nursing staff perfectly adhered to prescribed specimen acquisition protocols. In addition, we found that skin flora and common contaminants were present in low-density specimens. We do note that the hospital protocol at the University of Michigan dictates that nursing staff perform and informal quality control check by verifying the presence of fecal soilage after rectal swab collection, which we verified immediately prior to specimen processing. We also evaluated for systematic differences between nursing staff in different hospital units, and for significant differences between technically challenging rectal swabs acquired from mechanically ventilated patients and found no evidence of systematic bias in these indirect analyses. Despite this limitation, these rectal swab specimens predicted the onset of infection with both bacterial density and community composition. The differences in community composition between infected and uninfected patients were not driven by sequencing contaminants or skin flora but rather by enteric organisms, including gram-negative organisms that matched distal clinical isolates and known probiotic *Lactobacillus* bacteria [56, 57]. Given that the same set of rectal swabs predicted the onset of infection when characterized by both bacterial density and community composition, we believe that it is unlikely that the observed variation is due solely to the specimen acquisition technique.

Some studies have questioned the use of rectal swab specimens for the characterization of gut microbiota by demonstrating that temporally discordant fecal and rectal swab specimens have discordant gut microbiota [67]. Those results are inconsistent with other studies, which show concordance between rectal swab and fecal specimens in hematology-oncology patients [11], critically ill patients [74], and healthy outpatients [9, 10]. Our group and others have shown that gut microbiota undergo rapid and temporally dependent changes in hospitalized patients [15, 75, 76]; therefore, the finding that temporally discordant samples show large differences in observed microbiota is unsurprising. This study adds to the growing body of literature demonstrating the clinical utility of rectal swab specimens for the characterization of gut microbiota, and we replicate findings that admission rectal swabs are predictive of infection and outcomes in ICU patients [64, 77].

This retrospective cohort study using a convenience sample of rectal swabs has limitations that should prompt further validation and study. As a single-center study, our results may not be generalizable beyond the observed cohort. We quantified bacterial density using DNA quantification of individual timepoints, which cannot reliably distinguish living bacteria from dead bacteria or describe the dynamic variation in bacterial density throughout hospitalization. We also could not reliably record the mass of fecal material present on our rectal swab specimens or normalize the bacterial density of rectal swabs to the mass of fecal material on those swabs. Despite these limitations, which should obscure any clinically meaningful associations, we were able to detect significant associations between bacterial density, clinical covariates, and infection-free survival.

## Conclusions

Population density is a fundamental parameter in understanding the health and function of any ecosystem, but has largely been ignored in the study of the gut microbiome. Here, we demonstrate the methodological, biological, and clinical importance of bacterial density quantification. Our findings should prompt further study of this fundamental parameter of the gut microbial ecosystem.

## Supplementary Information

**Additional file 1: Supplemental Table 1.** Univariate comparisons of difference in bacterial density by demographics and comorbidities. **Supplemental Table 2.** Total antibiotic exposure in the cohort. **Supplemental Table 3.** Summary statistics of bacterial density by hospital unit. **Supplemental Table 4.** Tukey HSD comparisons of bacterial density by unit of admission. **Supplemental Table 5.** Alternative linear mixed effects model of features associated with bacterial density (log 16S copies/specimen) including unit of admission and mechanically ventilated status. **Supplemental Table 6.** Composite outcomes in the cohort. **Supplemental Table 7.** Pathogens isolated in cohort. **Supplemental Table 8.** Alternative multivariable frailty model of features associated with bacterial infection with unit of admission and mechanically ventilated status included. **Supplemental Table 9.** Features driving separation in community structure identified by random forest achieving significance after correcting for feature importance bias. **Supplemental Figure 1.** No relationship between unit of admission and bacterial density. We found no significant difference in bacterial density for patients admitted to different hospital units (*p*=0.33 by Kruskal-Wallis test).

**Additional file 2.**

## Data Availability

The dataset supporting the results of this article has been posted to the NIH Sequence Read Archive (accession number PRJNA633879). OTU tables, taxonomy classification tables, and metadata tables are available at https://github.com/rishichanderraj/RectalSwab_BacterialDensity. Protected health information is not included in our repository, but investigators who wish to build on our data may send reasonable requests that guarantee patient safety and privacy to gain access to this data.

## Abbreviations

EDTA: Ethylenediaminetetraacetic acid
pH: Potential of hydrogen
DNA: Deoxyribonucleic acid
cDNA: Complementary DNA
rRNA: Ribosomal ribonucleic acid
OTU: Operational taxonomic unit
PCR: Polymerase chain reaction
rpm: Revolutions per minute
ddPCR: Droplet digital PCR
V4: Fourth hypervariable region of the 16S gene
IQR: Interquartile range
SOFA: Sequential organ failure score
PERMANOVA: Permutational analysis of variance
PIMP: Permutation importance
VRE: Vancomycin-resistant enterococcus
