# Supplementary material for: The bacterial density of clinical rectal swabs is highly variable, correlates with sequencing contamination, and predicts patient risk of extraintestinal infection
Source: Microbiome. 2022 Jan 6;10:2. doi: 10.1186/s40168-021-01190-y (PMC8734160; doi:10.1186/s40168-021-01190-y)
Supplement: Supplementary file 2 — Additional file 1: Supplemental Table 1. Univariate comparisons of difference in bacterial density by demographics and comorbidities. Supplemental Table 2. Total antibiotic exposure in the cohort. Supplemental Table 3. Summary statistics of bacterial density by hospital unit. Supplemental Table 4. Tukey HSD comparisons of bacterial density by unit of admission. Supplemental Table 5. Alternative linear mixed effects model of features associated with bacterial density (log 16S copies/specimen) including unit of admission and mechanically ventilated status. Supplemental Table 6. Composite outcomes in the cohort. Supplemental Table 7. Pathogens isolated in cohort. Supplemental Table 8. Alternative multivariable frailty model of features associated with bacterial infection with unit of admission and mechanically ventilated status included. Supplemental Table 9. Features driving separation in community structure identified by random forest achieving significance after correcting for feature importance bias. Supplemental Figure 1. No relationship between unit of admission and bacterial density. We found no significant difference in bacterial density for patients admitted to different hospital units (p=0.33 by Kruskal-Wallis test). [file 40168_2021_1190_MOESM2_ESM.pdf]

Title: The bacterial density of clinical rectal swabs is highly variable, correlates with sequencing contamination, and predicts patient risk of extraintestinal infection

Authors: Rishi Chanderraj MD[1,2], Christopher A. Brown[2], Kevin Hinkle[2], Nicole Falkowski[2], Robert J. Woods[1,4], Robert P. Dickson[2,3,5]

Affiliations: 1. Division of Infectious Diseases, Department of Internal Medicine, University of Michigan Medical School, Ann Arbor, MI, USA. 2. Division of Pulmonary and Critical Care Medicine, Department of Internal Medicine, University of Michigan Medical School, Ann Arbor, MI, USA. 3. Department of Microbiology and Immunology, University of Michigan Medical School, Ann Arbor, MI, USA. 4. Computational Medicine and Bioinformatics, University of Michigan Medical School, Ann Arbor, MI, USA.5. Michigan Center for Integrative Research in Critical Care; Ann Arbor, MI, USA.

Methods

Study setting and design:

We designed a retrospective cohort study using a secondary analysis of clinically collected rectal swabs from hospitalized patients. We used hospital admission swabs previously collected, processed, and analyzed for a study of gut microbiome risk factors for Vancomycin resistant Enterococcus (VRE) acquisition in 118 patients admitted to the University of Michigan Hospital in 2016-2017[1]. In the prior study, used 236 rectal swab samples from 59 matched pairs to the study of gut microbiota of case and control subjects admitted to the University of Michigan Hospital during the study period. The infection control practice throughout the study period was to perform routine surveillance for VRE using rectal swabs on eight adult hospital units, including intensive care units, the hematology and oncology ward, and the bone marrow transplant ward. All hospitalized patients had routine collection of rectal swabs on admission and weekly thereafter to screen for VRE. In the prior study, cases were defined as subjects with an initial negative swab followed by a positive swab when evaluated by selective culture. We identified the "time at risk" for each case patient, defined as the time elapsed between admission and positive VRE screen. We matched each case subject to a control subject with an initial negative swab followed by repeat negative swab within the same time at risk (±5%). An additional matching factor was the unit from which the first positive VRE was recovered for cases or the matched swab after the time at risk for controls. For the current study, we restricted our analysis to admission rectal swabs (one swab per patient). We preformed an analysis on the entire cohort without reference to VRE colonization status.

Bacterial DNA isolation:

DNA isolation was performed with a single kit according to a modified protocol previously demonstrated to isolate bacterial DNA[5]. Briefly, rectal swab specimens were re-suspended in 360 µl ATL buffer (cell lysis solution, Qiagen DNeasy Blood & Tissue kit, catalog no. 69506) and homogenized in PowerBead Tubes (Qiagen, Hilden, Germany, catalog no. 13123-50).. ZymoBIOMICS Microbial Community DNA Standard (Zymo Research cat# D6306) was sequenced as a positive control. Sterile laboratory water, AE buffer (solution of 10 mM Tris-Cl 0·5 mM in EDTA; pH 9·0), and extraction control specimens were collected and analyzed as potential sources of contamination (negative controls).

Bacterial density quantification

Bacterial DNA quantification Bacterial DNA was quantified using a QX200 Droplet Digital PCR System (BioRad, Hercules, CA). The technique partitions a single sample into 20,000 droplets. A standard PCR reaction then amplifies 16S specific cDNA in each droplet, and each droplet is individually counted by the associated target dependent fluorescence signal as positive or negative. This allows for absolute 16S copy number quantification sample without generating a standard curve18–20. Primers and cycling conditions were performed according to a previously published protocol20. Specifically, primers were 5'- GCAGGCCTAACACATGCAAGTC-3' (63F) and 5'- CTGCTGCCTCCCGTAGGAGT-3' (355R). The cycling protocol was 1 cycle at 95°C for 5 minutes, 40 cycles at 95°C for 15 seconds and 60°C for 1 minute, 1 cycle at 4°C for 5 minutes, and 1 cycle at 90°C for 5 minutes all at a ramp rate of 2°C/second. The BioRad C1000 Touch Thermal Cycler was used for PCR cycling. Droplets were detected using the automated droplet reader (Bio-Rad, catalog no. 1864003), quantified using Quantasoft™ Analysis Pro (version 1.0.596), and imported to R for visualization and statistical analysis.

16s rRNA gene sequencing

The V4 region of the 16s rRNA gene was amplified using published primers and the dual-indexing sequencing strategy described previously[2]. Sequencing was performed using the Illumina MiSeq platform (San Diego, CA), using a MiSeq Reagent Kit V2 (500 cycles), according to the manufacturer's instructions with modifications found in the standard operating procedure of the laboratory of Dr. Patrick Schloss[3,4] Sequencing reagents were prepared according to the Schloss SOP and custom read 1, read 2, and index primers

were added to the reagent cartridge. Amplicons were sequenced using the Illumina MiSeq platform (San Diego, CA) using a MiSeq Reagent Kit V2 (Illumina, catalog no. MS102-2003) for 500 cycles. A synthetic community (n=4; ZymoBIOMICS Microbial Community DNA Standard, Zymo Research catalog no. D6306) was sequenced as a positive control. Sterile laboratory water (n=8), AE buffer (solution of 10 mM Tris-Cl 0·5 mM in EDTA; pH 9·0, [n=6]) used in DNA isolation, and extraction control specimens (n=6),were collected and analyzed as potential sources of contamination (negative controls). FASTQ files were generated with paired end reads and retained for further analysis.

Adequacy of sequencing.

We performed 16S rRNA gene amplicon sequencing on 236 rectal swab specimens and 15 negative-control specimens, which identified 1,188 unique operational taxonomic units (genus-level bacterial taxa) at a dissimilarity threshold of 3%. After bioinformatics processing, the mean number of reads per sample was 71,484 ± 2,684. No specimens were excluded from the analysis.

16S Gene analysis:

16S rRNA gene sequencing data were processed using mothur (v. 1.43.0) according to the Standard Operating Procedure for MiSeq sequence data using a minimum sequence length of 250 base pairs[4]. To summarize, the SILVA rRNA database[5] (v. 132, silva.nr_v132.regionV4.align) was used as a reference for sequence alignment and taxonomic classification. K-mer searching with 8-mers was used to assign raw sequences to their closest matching template in the reference database, and pairwise alignment was performed with the Needleman-Wunsch[6] and NAST algorithms[7]. A k-mer-based naive Bayesian classifier[8] was used to assign sequences to their correct taxonomy with a bootstrap confidence score threshold of 80. Pairwise distances between aligned sequences were calculated by the method employed by Sogin et al.[2] where pairwise distance equals mismatches, including indels, divided by sequence length. A distance matrix was passed to the OptiCLUST clustering algorithm[9] to cluster sequences into "operational taxonomic units" (OTUs) by maximizing the Matthews correlation coefficient with adissimilarity threshold of 3%[10]. OTU numbers were arbitrarily assigned in the binning process and are referred to throughout the manuscript in association with their most specified level of taxonomy (typically genus or family). OTUs were classified using the mothur implementation of the Ribosomal Database Project (RDP) classifier and RDP taxonomy training set 16 (trainset16_022016.rdp.fasta, trainset16_022016.rdp.tax), available on the mothur website[4]. After clustering and classification of raw sequencing data, we evaluated differences in community structure with permutational multivariate analysis of variance (PERMANOVA) in the vegan package (v2.0-4) [11] in R (v 3.6.4)[12]. We performed resampling of multiple generalized linear models with the *mvabund*[13] package in R to look for individual OTU differences between communities. We set a significance threshold of 0.01 after adjusting for multiple comparisons using a stepdown resampling procedure to reduce the type I error rate[14]. We confirmed individual OTU differences with random forest classification and regression models built with the ranger package in R (v 0.11.2)[15]. We used the caret (v 6.0-84)[16] package in R for cross-validation and to optimize the hyperparameters of the number of decision trees in the model and the number of features considered by each tree when splitting a node. We corrected for feature importance bias in random forest models with a permutation importance (PIMP) heuristic developed by Altmann et al.[17].

Clinical metadata:

We collected data from the electronic medical record to describe host health both by the severity of the acute illness that prompted hospitalization and by the severity of chronic disease before hospitalization. We measured acute illness and chronic disease with the validated Sequential Organ Failure Assessment Score (SOFA score)[18–20] and Charlson comorbidity index[21–23], respectively. We collected data on the antibiotic exposure of patients in the Emergency Department prior to collection of their initial rectal swab. 116 of the 118 rectal swabs in this cohort belonged to patients with accessible clinical metadata through the electronic medical record and were included in our analysis. 2 rectal swabs belonged to patients with sensitive information inaccessible through the electronic medical record and outside of the scope of our Institutional Research Board approval. Thus, only 116 of 118 subjects were included in the clinical metadata analysis.

We used infection-free survival to study the prognostic significance of bacterial density on rectal swabs. We defined extra-intestinal infection as the growth of a bacterial organism by traditional culture media in a site considered by clinicians to be "sterile" (blood, urine, ascites fluid, cerebrospinal fluid, sputum, deep tissue culture) meeting clinical criteria set by major medical societies and the Centers for Disease Control and Prevention for bacterial peritonitis[24], urinary tract infection[25,26], pneumonia[27–29], skin and soft tissue infection[30], and bacteremia[29]. Clinical adjudication of positive culture growth led to categorization as colonization without infection, contamination, or clinical infection.

We reviewed the admission history and physical documentation as well as the hospital discharge summary to determine the admitting diagnosis for patients in the cohort. We broadly classified admitting diagnoses into 7 categories: cardiopulmonary disorder (which included congestive heart failure, myocardial infarction, and respiratory failure not attributable to pneumonia, and post-operative ICU stay after major cardiac surgery); primary neurologic disorder (which included intracranial hemorrhage, ischemic stroke, or post-

operative recovery after major neurosurgery), Sepsis syndrome (defined as a presumed infection on admission requiring the use of antibiotics), gastrointestinal disruption (which included inflammatory bowel disease, pancreatitis, bowel obstruction or perforation, or post operative status after major gastrointestinal surgery), trauma, non-infectious complications of chemotherapy (which included acute renal injury, cytopenia without the presence of neutropenic fever, and nausea and vomiting attributable to chemotherapy), and non-infectious complications of bone-marrow transplantation (which included graft versus host disease as well as nausea and vomiting in the absence of recent chemotherapy administration)

Statistical analysis of clinical metadata:

All analyses were performed using the R programming statistical programming language (v 4·0·2)[12]. A multivariate linear regression model using clinical covariates to predict log transformed bacterial density was built with the *stats* package in R[12]. We constructed Kaplan-Meier curves to determine the median infection free survival in subjects above and below a critical threshold of 10^6 16S copies/sample. We used a stratified log-rank statistic to determine the statistical significance of differences in infection free survival between groups. After checking the proportional hazards assumption, we built Cox proportional hazards models incorporating bacterial density and clinical covariates were built to predict infection free survival. All survival analysis was done with the *survival*[31] (v 3·1-8) package in R .Pairwise significance was determined as appropriate by the Wilcoxon test with the Benjamini-Hochberg correction for multiple comparisons, Tukey's HSD test, and two-sample independent Mann-Whitney U test. All statistical tests used p=0.05 as a threshold for significance.

Appendix Table 1:	Univariate comparisons of difference in bacterial density by demographics and comorbidities
Appendix Table 2:	Total antibiotic exposure in the cohort
Appendix Table 3:	Summary statistics of bacterial density by hospital unit
Appendix Table 4:	Tukey HSD comparisons of bacterial density by unit of admission
Appendix Table 5:	Alternative linear mixed effects model of features associated with bacterial density (log 16S copies/specimen) including unit of admission and mechanically ventilated status
Appendix Table 6:	Composite outcomes in the cohort
Appendix Table 7:	Pathogens isolated in cohort
Appendix Table 8:	Alternative multivariable frailty model of features associated with bacterial infection with unit of admission and mechanically ventilated status included
Appendix Table 9:	Features driving separation in community structure identified by random forest achieving significance after correcting for feature importance bias
Supplemental Figure 1	Bacterial density by hospital unit

Appendix Table 1: Univariate comparisons of difference in bacterial density by demographics and comorbidities

	N=116	log(16S copies/sample)±SE		p-value
**Demographics**	**N (proportion)**	**Present**	**Absent**	
Age (mean ± SE)	60.0±1.37			
Female	52 (0.45)	14.94±0.48	15.17±0.42	0.71
Non-white race	17 (0.15)	14.46±0.80	15.17±0.34	0.42
**Diagnoses and comorbidities**				
C. difficile infection	15 (0.13)	16.45±1.73	14.86±0.65	0.11
Leukemia	30 (0.26)	14.76±0.65	15.17±0.36	0.58
Lymphoma	14 (0.12)	15.57±1.04	15.00±0.33	0.61
Bone marrow transplant	20 (0.17)	14.01±0.70	15.28±0.35	0.11
Solid organ malignancy	81 (0.70)	15.02±0.36	15.17±0.62	0.83
Metastatic malignancy	54 (0.47)	15.64±0.46	14.56±0.42	0.09
Diabetes	47 (0.41)	15.85±0.52	14.53±0.38	0.04[ǂ]
Coronary artery disease	18 (0.16)	16.31±0.52	14.83±0.35	0.03
Congestive heart failure	38 (0.33)	15.39±0.57	14.91±0.38	0.48
COPD	53 (0.46)	14.90±0.44	15.21±0.45	0.62
Peripheral vascular disease	7 (0.06)	16.23±1.10	14.99±0.33	0.31
End stage renal disease	46 (0.40)	15.63±0.50	14.70±0.40	0.15
Connective tissue disease	5 (0.04)	15.81±1.56	15.03±0.32	0.65
Peptic ulcer disease	16 (0.14)	14.72±0.69	15.12±0.35	0.61
Cirrhosis	12 (0.10)	14.55±1.06	15.12±0.33	0.62
Cerebrovascular disease	24 (0.21)	15.78±0.58	14.88±0.36	0.19
Hemiplegia	10 (0.09)	15.4±0.76	15.03±0.34	0.67
Dementia	4 (0.03)	17.59±0.72	14.97±0.32	0.03[ǂ]
Charlson Score (mean ± SE)	4.0±0.19			

ǂ Not significant after applying Benjamini-Hochberg Procedure

Appendix Table 2. Total antibiotic exposure in the cohort

	Number received
Vancomycin	35
Metronidazole	22
Piperacillin-tazobactam	20
Cefepime	18
Cefoxitin	4
Amoxicillin-clavulanate	2
Oral Vancomycin	2
Meropenem	1
**Total**	**104**

Appendix Table 3. Summary statistics of bacterial density by hospital unit

Hospital Unit	N (proportion)	16S rRNA gene copies/sample (log scale)				
**Unit**	**n**	**Mean**	**Median**	**Minimum**	**Maximum**	**IQR**
Oncology + BMT	41	14.58	14.08	9.42	21.90	6.83
Surgical ICU	35	16.01	16.71	9.53	21.09	3.80
Medical ICU	28	14.70	15.55	10.06	19.47	5.33
Trauma Burn ICU	4	14.63	15.11	10.03	18.27	4.28
Neuro ICU	4	15.57	17.10	10.76	17.33	1.75
Cardiac ICU	4	14.33	15.77	10.02	17.19	3.59

Appendix Table 4: Comparisons of mean bacterial density (log 16S copies/sample) between hospital units by Tukey's HSD test

Comparison	Difference in means, log(16S copies/sample)	Adjusted p-value
Medical ICU-Cardiac ICU	0.439 (-4.818 - 5.696)	1.000
Neuro ICU – Cardiac ICU	1.312 (-5.642 - 8.266)	0.994
Oncology BMT -Cardiac ICU	0.32 (-4.832 - 5.472)	1.000
Surgical ICU -Cardiac ICU	1.745 (-3.446 - 6.936)	0.925
Trauma/Burn ICU -Cardiac ICU	0.372 (-6.582 - 7.326)	1.000
Neuro ICU-Medical ICU	0.873 (-4.384 - 6.13)	0.997
Oncology BMT-Medical ICU	-0.119 (-2.53 - 2.292)	1.000
Surgical ICU-Medical ICU	1.306 (-1.188 - 3.799)	0.653
Trauma/Burn ICU -Medical ICU	-0.067 (-5.324 - 5.189)	1.000
Oncology BMT-Neuro ICU	-0.992 (-6.144 - 4.159)	0.993
Surgical ICU-Neuro ICU	0.433 (-4.758 - 5.623)	1.000
Trauma/Burn ICU -Neuro ICU	-0.94 (-7.895 - 6.014)	0.999
Surgical-Oncology BMT	1.425 (-0.838 - 3.688)	0.453
Trauma/Burn ICU -Oncology BMT	0.052 (-5.1 - 5.203)	1.000
Trauma/Burn ICU -Surgical ICU	-1.373 (-6.564 - 3.818)	0.972

Appendix Table 5. Alternative linear mixed effects model of features associated with bacterial density (log 16S copies/specimen) including unit of admission and mechanically ventilated status

	Coefficient (95% CI)	P value
Piperacillin-tazobactam	-2.092 (-3.464–0.713)	0.006**
Age (decade)	0.044 (0.005-0.084)	0.040
Charlson comorbidity index	0.401 (0.122-0.674)	0.008
SOFA Score	-0.018 (-0.239-0.204)	0.882
VRE colonization	0.204 (-0.875-1.29)	0.725
Mechanical Ventilation	1.117 (-0.235-2.434)	0.121
Unit of admission (relative to Oncology BMT ward)		
Neuro ICU	1.201 (-1.802-4.19)	0.455
Trauma/Burn ICU	-1.05 (-4.305-2.242)	0.551
Surgical ICU	0.825 (-0.599-2.247)	0.285
Medical ICU	-0.623 (-2.234-0.978)	0.472
Cardiac ICU	-0.474 (-3.52-2.531)	0.771
REML criteria at convergence: 584.7		

Appendix Table 6. Composite outcomes in cohort

	Number
Bacteremia	14
Pneumonia	10
Urinary tract infection	8
Soft tissue infection	3
Bacterial Peritonitis	2
**Total**	**37**

Appendix Table 7. Pathogens isolated in cohort

Organism	Bacteremia (% blood culture)	Pneumonia (% respiratory culture)	Urinary (% urine culture)	Soft tissue (% tissue culture)	Peritonitis (% ascites culture)	Total (% of all cultures)
Staphylococcus aureus	2	5	0	2	0	9
Escherichia coli	0	1	3	0	0	4
Pseudomonas aeruginosa	1	2	1	0	0	4
Enterococcus faecalis	3	0	0	0	0	3
Enterobacter aerogenes	1	0	1	0	0	2
Enterococcus faecium	1	0	1	0	0	2
Klebsiella pneumoniae	1	0	1	0	0	2
Acinetobacter baumannii	0	1	0	0	0	1
Bacteroides thetaiotaomicron	1	0	0	0	0	1
Clostridium perfringens	0	0	0	1	0	1
Corynebacterium striatum	0	1	0	0	0	1
Enterobacter cloacae	0	0	1	0	0	1
Klebsiella oxytoca	1	0	0	0	0	1
Streptococcus anginosus	0	0	0	0	1	1
Streptococcus Group B	0	0	0	0	1	1
Streptococcus Group G	1	0	0	0	0	1
Streptococcus pneumoniae	1	0	0	0	0	1
Streptococcus salivarius	1	0	0	0	0	1
Contamination	1	1	13	2	1	18
No growth	90	25	68	13	7	203
**Total culture type**	**105**	**36**	**89**	**18**	**10**	**258**

Appendix Table 8. Alternative multivariable frailty model of features associated with bacterial infection with unit of admission and mechanically ventilated status included

	Hazard ratio (95% CI)	P value
log(copies 16S/sample)	1.198 (1.037-1.384)	0.014**
SOFA Score	0.926 (0.782-1.097)	0.376
Charlson Comorbidity Index	1.032 (0.957-1.112)	0.420
VRE colonization	0.575 (0.267-1.237)	0.157
Piperacillin-tazobactam	1.57 (0.53-4.648)	0.416
Admission diagnosis of sepsis	2.418 (0.909-6.429)	0.077
Mechanically ventilated	2.08 (0.748-5.785)	0.161
Unit of admission (relative to Oncology BMT ward)		
Neuro ICU	1.142 (0.072-18.1)	0.925
Trauma_burn ICU	1.545 (0.094-25.337)	0.761
Cardiac ICU	1.953 (0.095-40.038)	0.664
Medical ICU	4.082 (0.884-18.851)	0.072
Surgical ICU	2.344 (0.611-8.998)	0.215
Number of events = 37	Likelihood ratio test: $p < 2*10^{-8}$	Concordance: 0.923

Appendix Table 9. Features driving separation in community structure identified by random forest achieving significance after correcting for feature importance bias

features	Mean Decrease in Accuracy	pvalue	Genus
Otu0054	3.60E-03	2.97E-02	Megasphaera
Otu0026	2.64E-03	4.95E-02	Lactobacillus
Otu0051	2.34E-03	1.98E-02	Lachnospiracea_incertae_sedis
Otu0002	1.69E-03	4.95E-02	Enterobacteriaceae_unclassified
Otu0031	1.55E-03	3.96E-02	Clostridium_XlVa
Otu0013	1.51E-03	3.96E-02	Bacteroides
Otu0024	1.44E-03	4.95E-02	Parabacteroides
Otu0032	1.38E-03	1.98E-02	Parabacteroides
Otu0025	1.01E-03	1.98E-02	Clostridiales_unclassified
Otu0045	8.90E-04	3.96E-02	Bacteroides
Otu0061	7.38E-04	2.97E-02	Lachnospiraceae_unclassified
Otu0062	7.16E-04	4.95E-02	Streptococcus
Otu0070	5.86E-04	4.95E-02	Clostridium_XlVa
Otu0113	4.35E-05	2.97E-02	Clostridiales_unclassified
Otu0189	-2.49E-04	2.97E-02	Actinomyces

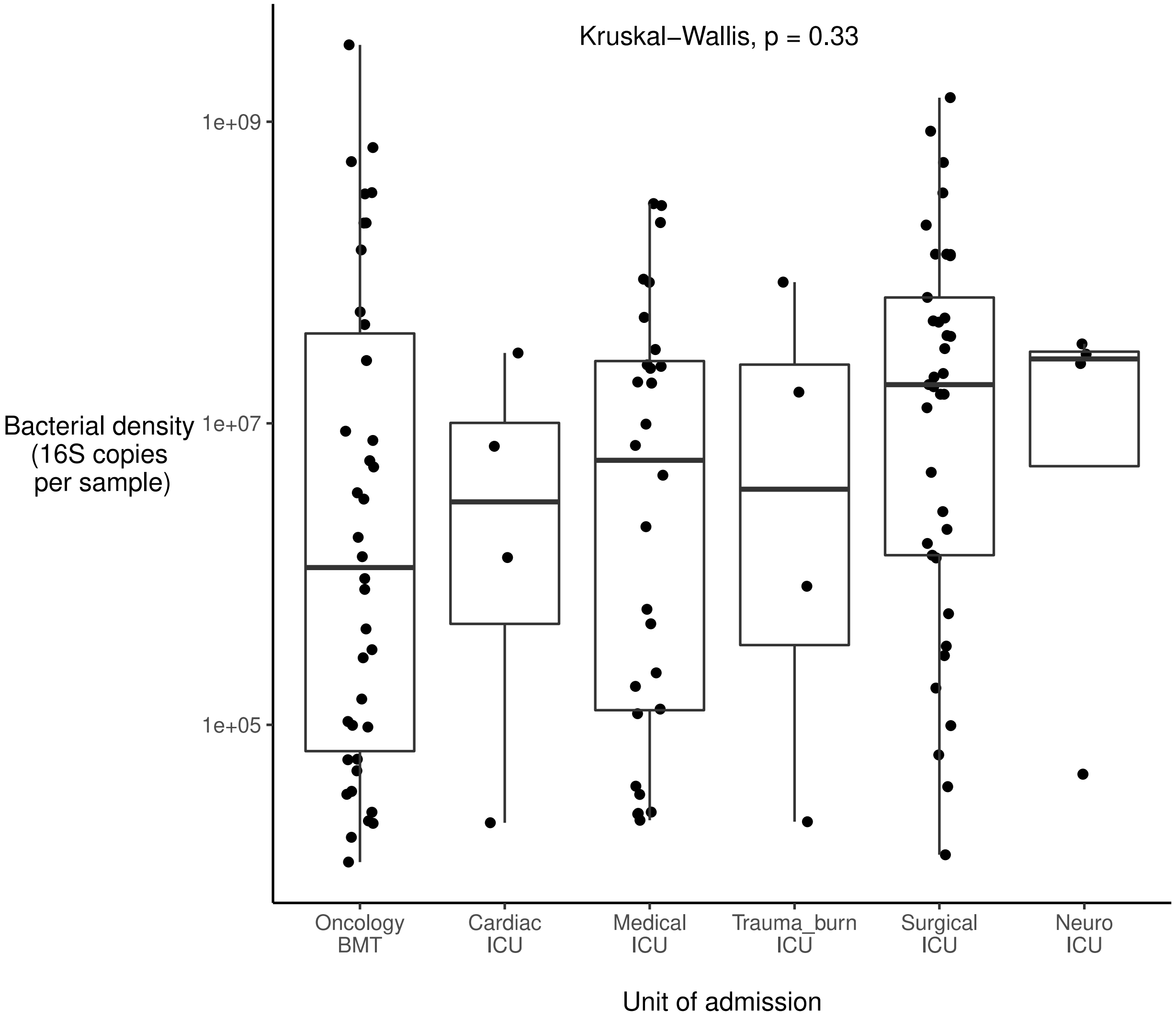

Supplemental Figure 1. No relationship between unit of admission and bacterial density. We found no significant difference in bacterial density for patients admitted to different hospital units (p=0.33 by Kruskal-Wallis test).

References

1 Chanderraj R, Brown CA, Hinkle K, *et al.* Gut Microbiota Predict *Enterococcus* Expansion but Not Vancomycin-Resistant *Enterococcus* Acquisition. *mSphere* 2020; **5**. DOI:10.1128/mSphere.00537-20.

2 Sogin ML, Morrison HG, Huber JA, *et al.* Microbial diversity in the deep sea and the underexplored 'rare biosphere'. *Proc Natl Acad Sci U S A* 2006; **103**: 12115–20.

3 Kozich JJ, Westcott SL, Baxter NT, Highlander SK, Schloss PD. Development of a dual-index sequencing strategy and curation pipeline for analyzing amplicon sequence data on the miseq illumina sequencing platform. *Appl Environ Microbiol* 2013; **79**: 5112–20.

4 Schloss PD. MiSeq SOP:mothur. MiSeq SOP:mothur. 2019. http://www.mothur.org/wiki/MiSeq_SOP (accessed Feb 11, 2019).

5 Quast C, Pruesse E, Yilmaz P, *et al.* The SILVA ribosomal RNA gene database project: improved data processing and web-based tools. *Nucleic Acids Res* 2012; **41**: D590–6.

6 Needleman SB, Wunsch CD. A general method applicable to the search for similarities in the amino acid sequence of two proteins. *J Mol Biol* 1970; **48**: 443–53.

7 Caporaso JG, Bittinger K, Bushman FD, DeSantis TZ, Andersen GL, Knight R. PyNAST: a flexible tool for aligning sequences to a template alignment. *Bioinformatics* 2010; **26**: 266–7.

8 Wang Q, Garrity GM, Tiedje JM, Cole JR. Naïve Bayesian classifier for rapid assignment of rRNA sequences into the new bacterial taxonomy. *Appl Environ Microbiol* 2007; **73**: 5261–7.

9 Westcott SL, Schloss PD. OptiClust, an Improved Method for Assigning Amplicon-Based Sequence Data to Operational Taxonomic Units. *mSphere*; **2**. DOI:10.1128/mSphereDirect.00073-17.

10 Matthews BW. Comparison of the predicted and observed secondary structure of T4 phage lysozyme. *BBA - Protein Struct* 1975; **405**: 442–51.

11 Oksanen J, Blanchet FG, Kindt R, *et al.* Vegan: Community Ecology Package. R package version 2.0-2. 2012; published online Jan 1.

12 R Development Core Team R. R: A Language and Environment for Statistical Computing. 2019 DOI:10.1007/978-3-540-74686-7.

13 Wang Y, Naumann U, Wright ST, Warton DI. Mvabund- an R package for model-based analysis of multivariate abundance data. *Methods Ecol Evol* 2012; **3**: 471–4.

14 ter Braak CJF, Peres-Neto P, Dray S. A critical issue in model-based inference for studying trait-based community assembly and a solution. *PeerJ* 2017; **2017**. DOI:10.7717/peerj.2885.

15 Wright MN, Ziegler A. Ranger: A fast implementation of random forests for high dimensional data in C++ and R. *J Stat Softw* 2017; **77**. DOI:10.18637/jss.v077.i01.

16 Kuhn M. Building Predictive Models in *R* Using the **caret** Package. *J Stat Softw* 2008; **28**: 1–26.

17 Altmann A, Toloşi L, Sander O, Lengauer T. Permutation importance: a corrected feature importance measure. *Bioinformatics* 2010; **26**: 1340–7.

18 Cárdenas-Turanzas M, Ensor J, Wakefield C, *et al.* Cross-validation of a Sequential Organ Failure Assessment score–based model to predict mortality in patients with cancer admitted to the intensive care unit. *J Crit Care* 2012; **27**: 673–80.

19 Ferreira FL, Bota DP, Bross A, Mélot C, Vincent J-L. Serial Evaluation of the SOFA Score to Predict Outcome in Critically Ill Patients. *JAMA* 2001; **286**: 1754–8.

20 Vincent J-L, de Mendonca A, Cantraine F, *et al.* Use of the SOFA score to assess the incidence of organ dysfunction/failure in intensive care units: Results of a multicenter, prospective study. *Crit Care Med* 1998; **26**. https://journals.lww.com/ccmjournal/Fulltext/1998/11000/Use_of_the_SOFA_score_to_assess_the_incidence_of.16.aspx.

21 Radovanovic D, Seifert B, Urban P, *et al.* Validity of Charlson Comorbidity Index in patients hospitalised with acute coronary syndrome. Insights from the nationwide AMIS Plus registry 2002-2012. *Heart* 2014; **100**: 288–94.

22 Charlson ME, Pompei P, Ales KL, MacKenzie CR. A new method of classifying prognostic comorbidity in

longitudinal studies: Development and validation. *J Chronic Dis* 1987; **40**: 373–83.

23 Quan H, Li B, Couris CM, *et al.* Updating and validating the charlson comorbidity index and score for risk adjustment in hospital discharge abstracts using data from 6 countries. *Am J Epidemiol* 2011; **173**: 676–82.

24 Runyon BA. Introduction to the revised American Association for the Study of Liver Diseases Practice Guideline management of adult patients with ascites due to cirrhosis 2012. *Hepatology* 2013; **57**: 1651–3.

25 Hooton TM, Bradley SF, Cardenas DD, *et al.* Diagnosis, prevention, and treatment of catheter-aassociated urinary tract infection in adults: 2009 international clinical practice guidelines from the infectious diseases society of America. *Clin Infect Dis* 2010; **50**: 625–63.

26 Gupta K, Hooton TM, Naber KG, *et al.* International clinical practice guidelines for the treatment of acute uncomplicated cystitis and pyelonephritis in women: A 2010 update by the Infectious Diseases Society of America and the European Society for Microbiology and Infectious Diseases. *Clin Infect Dis* 2011; **52**: 103–20.

27 Klompas M, Kleinman K, Khan Y, *et al.* Rapid and reproducible surveillance for ventilator-associated pneumonia. *Clin Infect Dis* 2012; **54**: 370–7.

28 CDC, Oid, Ncezid, DHQP. Pneumonia (Ventilator-associated [VAP] and non-ventilator-associated Pneumonia [PNEU]) Event. 2020.

29 Horan TC, Andrus M, Dudeck MA. CDC/NHSN surveillance definition of health care-associated infection and criteria for specific types of infections in the acute care setting. *Am J Infect Control* 2008; **36**: 309–32.

30 Stevens DL, Bisno AL, Chambers HF, *et al.* Practice guidelines for the diagnosis and management of skin and soft tissue infections: 2014 update by the infectious diseases society of America. Clin. Infect. Dis. 2014; **59**: e10–52.

31 Therneau T. A Package for Survival Analysis in S. 2015.
