# Supplementary material for: The bacterial density of clinical rectal swabs is highly variable, correlates with sequencing contamination, and predicts patient risk of extraintestinal infection
Source: Microbiome. 2022 Jan 6;10:2. doi: 10.1186/s40168-021-01190-y (PMC8734160; doi:10.1186/s40168-021-01190-y)
Supplement: Supplementary file 3 — Additional file 2. [file 40168_2021_1190_MOESM3_ESM.pdf]

The bacterial density of clinical rectal swabs is highly variable, correlates with sequencing contamination, and predicts patient risk of extraintestinal infection

Rishi Chanderraj

8/14/2020

```
# For ease of use, place the processed 16S data files of interest into the same directory as your curre

# Basic processing with selection of OTUs
# I. Read in opti_mcc.shared
otu_good <- load_shared(shared = "vre.miseq.trim.contigs.good.unique.good.filter.unique.precluster.pick


# Trim _S from end of rownames(otu_good)
rownames(otu_good) <- str_remove(rownames(otu_good), "_S\\d+")


# II. Read in cons.taxonomy
otu_good_taxonomy <- load_tax("vre.miseq.trim.contigs.good.unique.good.filter.unique.precluster.pick.pi
```

```
# Create lables to check for real specimen
otu_df <- data.frame(decostand(otu_good, "total") * 100, Sample_name = row.names(otu_good), stringsAsFa
  # Create Experiment from first string of characters prior to first "_"
  mutate(experiment = factor(case_when(str_detect(Sample_name, "Woods_") ~ "Swab",
                                       str_detect(Sample_name, "^AE_") ~ "AE",
                                       str_detect(Sample_name, "^Empty_") ~ "Empty",
                                       str_detect(Sample_name, "^IsoCtrl_") ~ "IsoCtrl",
                                       str_detect(Sample_name, "^Water|^WATER") ~ "Water",
                                       str_detect(Sample_name, "^Zymo") ~ "Mock"),
                             levels = c("Swab", "AE", "Empty", "IsoCtrl", "Water", "Mock"))
         ) %>%
  # metadata columns at front, followed by all of the count data
    mutate(experiment = str_extract(Sample_name, "^[:alpha:]+"))%>%
  mutate(Sample_ID = str_replace(Sample_name, "^[:alpha:]+_","")) %>%
  mutate(experiment = if_else(experiment == "Woods",
                              str_replace(experiment, "Woods", "Swab"),
                              experiment))%>%
  dplyr::select(Sample_ID, experiment,everything(),-Sample_name)
  # otu_df with rownames added for PCA analysis later
  # note that the data is in wide format
```

```
# gather the OTU from wide to long
tidy_otu_df<-otu_df %>%
  gather("Otu", "relative_abundance", -c(Sample_ID, experiment))%>%
```

```
  mutate(Otu = as_factor(Otu))

# link OTU to genus
otu_genus_link <- otu_good_taxonomy%>%
  dplyr::select(OTU,Genus)%>%
  mutate(Otu_genus = str_c(OTU,Genus, sep="-"))%>%
  rename("Otu"=OTU)%>%
  mutate(Otu = as_factor(Otu))# first create a new variable

tidy_genus_df<-inner_join(tidy_otu_df, otu_genus_link, by = "Otu")
rm(tidy_otu_df)
```

```
# this file is the results from Nicole for the ddPCR runs on the stoll swab specimens
ddPCR<- read_excel("16S_EvaGreen_WoodsRectalSwabs_Combined.xlsx",
    col_types = c("text", "text", "skip",
        "skip", "skip", "skip", "skip",
        "skip", "numeric", "skip", "skip",
        "skip", "skip", "skip", "skip",
        "skip"), sheet = 1)%>%
  mutate(Sample_ID = str_replace(Sample, "^Iso[:space:]Ctrl[:space:]", ""))%>%
  rename(swab_type = `Case/Control`)%>%
  mutate(swab_type = if_else(swab_type=="Case","case",swab_type),
         swab_type = if_else(swab_type=="Control","control",swab_type))%>%
  mutate(experiment = case_when(swab_type == "IsoCtrl" ~ "Isolation control",
                                swab_type == "Water" ~ "Water",
                                TRUE ~ "Rectal swab"))%>%
  rename(ddPCR_reads_per_sample = `Total 16S copies/isolation`)%>%
  filter(!is.na(ddPCR_reads_per_sample))%>%
  dplyr::select(Sample_ID, swab_type, experiment, ddPCR_reads_per_sample)%>%
  mutate(Sample_ID = if_else(experiment=="Water","NEG", Sample_ID))%>%
  filter(!(ddPCR_reads_per_sample>18 & experiment=="Water"))%>%
  mutate(Sample_ID = if_else(experiment=="Water",paste(Sample_ID,LETTERS[1:25],sep=""), Sample_ID))# dp
# remove duplicate "Sample" variable, order variables to join
summary(ddPCR$ddPCR_reads_per_sample)
```

```
##      Min.   1st Qu.    Median      Mean   3rd Qu.      Max.
## 9.000e+00 4.975e+04 1.151e+06 8.532e+07 3.623e+07 3.234e+09
```

```
cases<-ddPCR %>% filter(swab_type == "case")%>%dplyr::pull(Sample_ID)%>%as_factor() # create a vector o
control<-ddPCR %>% filter(swab_type == "control")%>%dplyr::pull(Sample_ID)%>%as_factor()

# this file is the results from Miseq summary
Miseq_quant <- read_csv("workbook.csv")[, 1:2]%>%
  rename(Sample_ID = `Sample ID`, miseq_reads_per_sample = `Reads PF/Sample`)%>%
  mutate(experiment = str_extract(Sample_ID, "^[:alpha:]+"))%>%
  mutate(Sample_ID = str_replace(Sample_ID, "^[:alpha:]+_","")) %>%
  mutate(experiment = if_else(experiment == "Woods",
                              "Rectal swab",
                              experiment))%>%
  mutate(swab_type = as_factor(case_when(Sample_ID %in% cases ~ "Case",
                                Sample_ID %in% control ~ "Control",
                                TRUE ~ "Isolation Control")))%>%
  dplyr::select(Sample_ID, swab_type, experiment, miseq_reads_per_sample)
```

```
## Warning: Missing column names filled in: 'X3' [3]
```

```r
ddPCR_miseq_comparison<-left_join(ddPCR, Miseq_quant, by = "Sample_ID")%>%
  mutate(experiment.y = if_else(experiment.y== "AE", "Elution buffer", experiment.y),
         experiment.y = if_else(experiment.y=="IsoCtrl","Isolation control",experiment.y),
         experiment.y =factor(experiment.y, levels=c("Water","Isolation control","Elution buffer", "Rec
  mutate(ddPCR_reads_per_sample=if_else(experiment.y=="Elution buffer", 0,
                                        ddPCR_reads_per_sample))%>%
  mutate(ddPCR_reads_per_sample = na_if(ddPCR_reads_per_sample, 0))

# Table 1 ddPCR
ddPCR_miseq_comparison%>%
  group_by(experiment.x)%>%
  summarize(`Mean` = mean(ddPCR_reads_per_sample,na.rm=T),
            `Median` = median(ddPCR_reads_per_sample,na.rm=T),
            `Minimum` = min(ddPCR_reads_per_sample,na.rm=T),
            `Maximum` = max(ddPCR_reads_per_sample,na.rm=T),
             `Standard deviation` = sd(ddPCR_reads_per_sample,na.rm=T),
            `Interquartile range` = IQR(ddPCR_reads_per_sample,na.rm=T))%>%
  mutate_if(is.double,~round(.,digits=2))%>%
  pivot_longer(cols=-experiment.x, names_to="metric", values_to="value")%>%
  pivot_wider(names_from=experiment.x, values_from=value)%>%
  dplyr::select(metric, Water, `Isolation control`, `Rectal swab`)
```

```
## # A tibble: 6 x 4
##   metric              Water `Isolation control` `Rectal swab`
##   <chr>               <dbl>               <dbl>         <dbl>
## 1 Mean                13.4                3150.     96625320.
## 2 Median              13.6                3223.      3165086.
## 3 Minimum             12.2                1408.        11846.
## 4 Maximum             14.4                5209.   3234306771.
## 5 Standard deviation   1.1                1278.    341147947.
## 6 Interquartile range  1.09                984.     46339173.
```

```r
# Table 1 miseq
ddPCR_miseq_comparison%>%
  group_by(experiment.x)%>%
  summarize(`Mean` = mean(miseq_reads_per_sample,na.rm=T),
            `Median` = median(miseq_reads_per_sample,na.rm=T),
            `Minimum` = min(miseq_reads_per_sample,na.rm=T),
            `Maximum` = max(miseq_reads_per_sample,na.rm=T),
             `Standard deviation` = sd(miseq_reads_per_sample,na.rm=T),
            `Interquartile range` = IQR(miseq_reads_per_sample,na.rm=T))%>%
  mutate_if(is.double,~round(.,digits=2))%>%
  pivot_longer(cols=-experiment.x, names_to="metric", values_to="value")%>%
  pivot_wider(names_from=experiment.x, values_from=value)%>%
  dplyr::select(metric, Water, `Isolation control`, `Rectal swab`)
```

```
## # A tibble: 6 x 4
##   metric               Water `Isolation control` `Rectal swab`
##   <chr>                <dbl>               <dbl>         <dbl>
## 1 Mean                73858.              53687.        73926.
## 2 Median              84455.              55708.        74505.
```

```
## 3 Minimum             44181.              23946.          83.7
## 4 Maximum             92939.              78621.      145353.
## 5 Standard deviation  26049.              17298.       22790.
## 6 Interquartile range 24379.              20939.       29514.
```

```
# Statistical testing
TukeyHSD(aov(log(ddPCR_reads_per_sample)~experiment.y,data=ddPCR_miseq_comparison))
```

```
##   Tukey multiple comparisons of means
##     95% family-wise confidence level
##
## Fit: aov(formula = log(ddPCR_reads_per_sample) ~ experiment.y, data = ddPCR_miseq_comparison)
##
## $experiment.y
##                                    diff         lwr      upr     p adj
## Isolation control-Water        5.388694 -0.04311471 10.82050 0.0523715
## Rectal swab-Water             12.242045  7.77865241 16.70544 0.0000000
## Rectal swab-Isolation control  6.853351  3.67734340 10.02936 0.0000022
```

```
TukeyHSD(aov(miseq_reads_per_sample~experiment.y,data=ddPCR_miseq_comparison))
```

```
##   Tukey multiple comparisons of means
##     95% family-wise confidence level
##
## Fit: aov(formula = miseq_reads_per_sample ~ experiment.y, data = ddPCR_miseq_comparison)
##
## $experiment.y
##                                          diff        lwr      upr     p adj
## Isolation control-Water          -24909.36441 -66290.248 16471.52 0.4052023
## Elution buffer-Water             -15434.03756 -56814.921 25946.85 0.7695710
## Rectal swab-Water                    68.10432 -33935.141 34071.35 0.9999999
## Elution buffer-Isolation control   9475.32685 -24312.023 43262.68 0.8868640
## Rectal swab-Isolation control     24977.46873    781.845 49173.09 0.0400965
## Rectal swab-Elution buffer        15502.14188  -8693.482 39697.77 0.3486703
```

```
just_swabs_otu<-otu_df %>%
  filter(experiment == "Swab")%>%
  dplyr::select(Sample_ID, everything())

all_data<-inner_join(ddPCR%>%dplyr::select(-experiment), otu_df)


swab_labels <- read_excel("case_control_samples_5_28_2019.xlsx") %>%
  unite("swab_label", c(swab_type, case_or_control), sep = "_", remove = FALSE)

ddPCR_labeled<-ddPCR %>% dplyr::select(-swab_type, -experiment)%>%
  inner_join(swab_labels, ddPCR, by = c("Sample_ID"))

all_data_swabs<-inner_join(ddPCR_labeled, just_swabs_otu, by =("Sample_ID"))
all_data_swabs<-all_data_swabs %>%
  mutate(above_threshold = as_factor(if_else(ddPCR_reads_per_sample >= 1e06, "above", "below")))
```

```r
# Make all our character columns factor
all_data_swabs<-all_data_swabs %>%
  mutate(swab_label = as_factor(swab_label)) %>%
  mutate(swab_type = as_factor(swab_type)) %>%
  mutate(case_or_control = as_factor(case_or_control))%>%
  mutate(shannon=diversity(.[,8:(nrow(.)-1)]))

all_data_swabs<-as.data.frame(all_data_swabs) # Hadley Wickham won't let you set rownames!  Gotta chang
rownames(all_data_swabs) <- all_data_swabs$Sample_ID
  # all_data_swabs_df with rownames added for PCA analysis later
  # note that the data is in wide format

all_data_swabs_tidy<-all_data_swabs%>%gather("Otu", "relative_abundance", -c(Sample_ID,ddPCR_reads_per_s
  mutate(Otu = as_factor(Otu))

# link OTU to genus
otu_genus_link <- otu_good_taxonomy%>%
  dplyr::select(OTU,Genus)%>%
  mutate(Otu_genus = str_c(OTU,Genus, sep="-"))%>%
  rename("Otu"=OTU)%>%
  mutate(Otu = as_factor(Otu))# first create a new variable

all_data_swabs%>%
  dplyr::select(above_threshold, Otu0001)%>%
  mutate(contaminant_present = as.numeric(Otu0001>0))%>%
  # group_by(above_threshold)%>%
  summarize(percent_contaminated = mean(contaminant_present))
```

```
##   percent_contaminated
## 1            0.6239316
```

```r
swab_initial<-all_data_swabs%>%
  filter(swab_type =="initial")%>%
  pull(Sample_ID)

samples_for_first_plot<-c(swab_initial,"1","2","3","4","5","6","NEGP","NEGQ","NEGR","NEGS","NEGT","NEGU
"NEGC","NEGD","NEGE","NEGF","NEGG","NEGH","NEGI","NEGJ","NEGK","NEGL","NEGM","NEGN","NEGO")

correlation_ddPCR_miseq<-ddPCR_miseq_comparison %>%
  filter(!is.na(experiment.y), Sample_ID %in% samples_for_first_plot)%>%
  ggplot(aes(x=ddPCR_reads_per_sample, y =miseq_reads_per_sample))+
    geom_point(aes(color= experiment.x),alpha=0.5)+
    scale_x_log10()+
    scale_y_log10()+
    labs(x="ddPCR: 16S gene copies per sample",
         y ="Illumina MiSeq 16S reads per sample") +
    coord_cartesian(ylim = c(10, max(Miseq_quant$miseq_reads_per_sample)))+
  theme_bw()+
  theme(panel.grid=element_blank(),
        legend.position = c(0.12,0.3),
        panel.border = element_blank(),
        axis.line = element_line())+
```

```
  scale_color_manual(values=c("#33a02c","#654321","#1f78b4"))+
  labs(color = NULL,cor=NULL)+
  stat_cor(label.y=4,method="pearson")+
  coord_cartesian(ylim=c(10^1.9,10^5.25))

correlation_ddPCR_miseq
```

```
## Warning: Removed 6 rows containing non-finite values (stat_cor).

## Warning: Removed 6 rows containing missing values (geom_point).
```

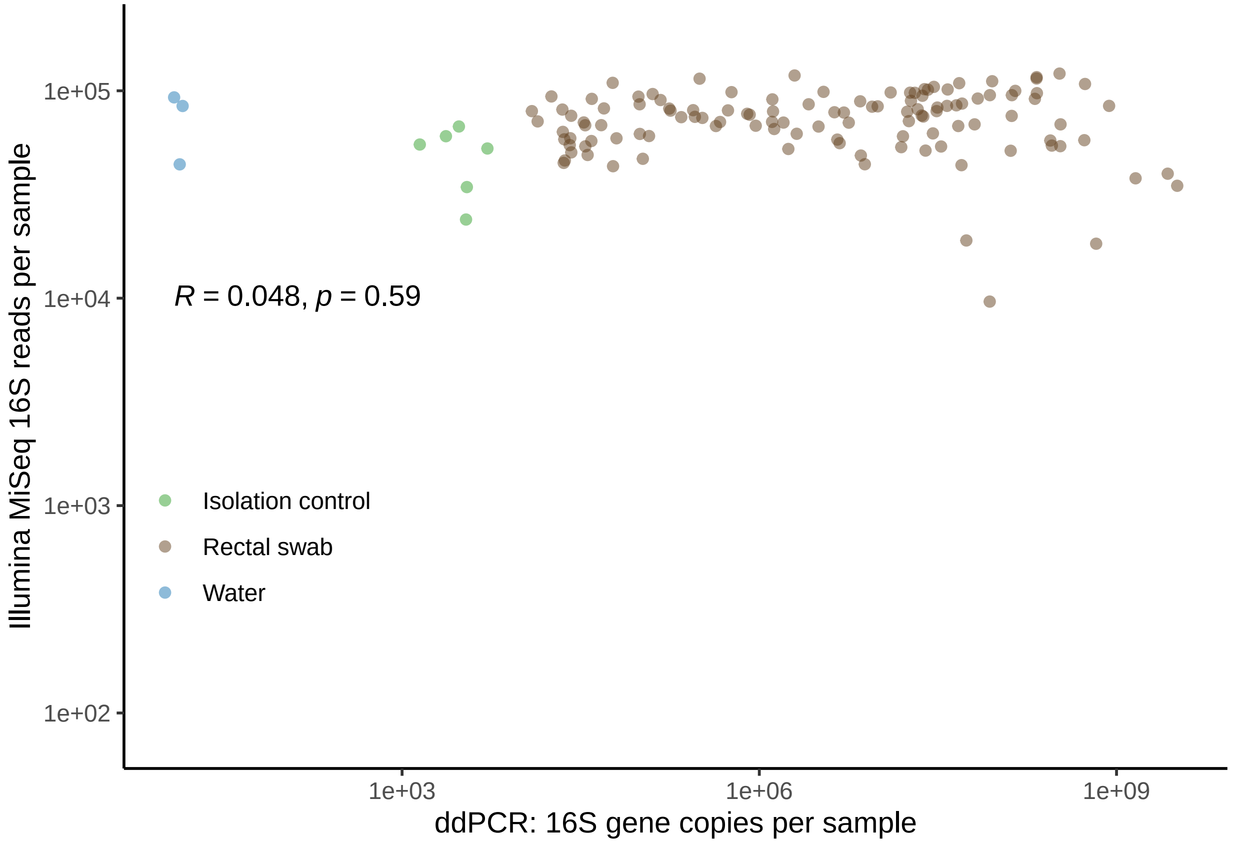


```
comparisons<-list(c("Isolation\ncontrol","Rectal\nswab"),
                  c("Water","Rectal\nswab"))

negative_control_v_sample<-ddPCR_miseq_comparison %>%
  pivot_longer(cols=c(ddPCR_reads_per_sample, miseq_reads_per_sample),
               names_to="read_type",
               values_to="read_count")%>%
  filter(experiment.y != "Elution buffer")%>%
  mutate(experiment.x = case_when(experiment.y=="Isolation control"~"Isolation\ncontrol",
                                  experiment.y=="Rectal swab"~"Rectal\nswab",
                                  experiment.y=="Water"~"Water",
                                  is.na(experiment.y)~"Water"),
         experiment.x=factor(experiment.x, levels=c("Water","Isolation\ncontrol",
```

```
                                                    "Rectal\nswab")),
         read_type = if_else(read_type=="ddPCR_reads_per_sample",
                             "ddPCR: 16S gene copies per sample",
                             "Ilumina MiSeq: 16S reads per sample"))%>%
  dplyr::select(-swab_type.x,-swab_type.y,-experiment.y)%>%
  rename(experiment = "experiment.x")


negative_control_v_sample<-negative_control_v_sample%>%
  ggplot(aes(x=experiment, y = read_count, color=experiment))+
  ggbeeswarm::geom_quasirandom(alpha=0.5)+
  facet_wrap(~read_type,scales = "free")+
  scale_y_log10()+
  theme_bw()+
  theme(panel.grid=element_blank(),
        legend.position = "none")+
  labs(x=NULL, y ="16S copies")+
  scale_color_manual(values=c("#a6cee3","#33a02c","#654321"))+
  ggpubr::stat_compare_means(comparisons=comparisons, label = "p.signif",method="wilcox.test",
                             na.rm = T)

negative_control_v_sample
```

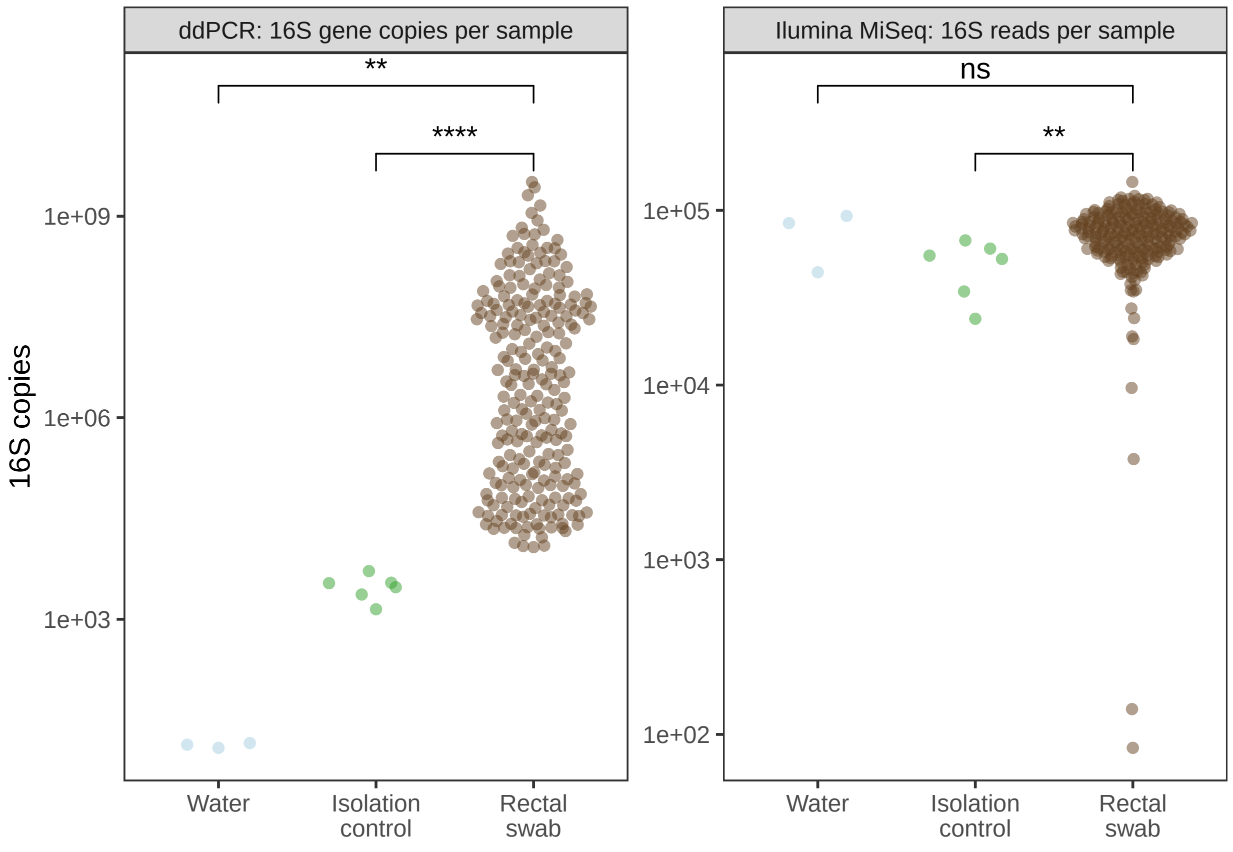


```
ddpcr_v_miseq<-ggarrange(negative_control_v_sample,
          correlation_ddPCR_miseq,
```

```
        nrow=2, align="h",labels = c("A", "B"))
```

```
## Warning: Removed 6 rows containing non-finite values (stat_cor).

## Warning: Removed 6 rows containing missing values (geom_point).

## Warning: Graphs cannot be horizontally aligned unless the axis parameter is set.
## Placing graphs unaligned.
```

```
# ddpcr_v_miseq
# ggexport(ddpcr_v_miseq, filename="ddpcr_v_miseq.pdf")
ddpcr_v_miseq
```

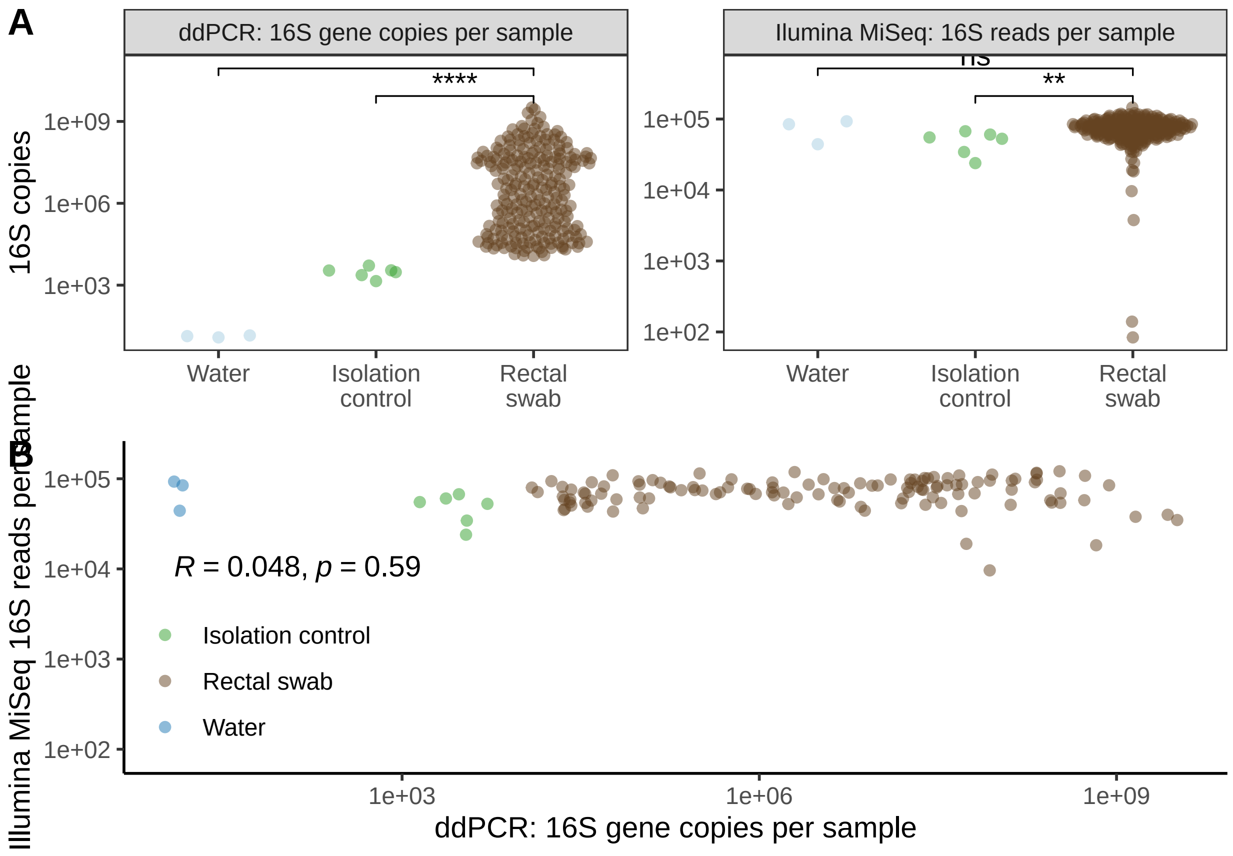


```
contaminant_abundance<-tidy_genus_df %>%
  filter(experiment %in% c("AE", "Empty", "WaterNeg","IsoCtrl") , relative_abundance >1) %>%
  group_by(Otu_genus)%>%
  summarize(mean_abundance=mean(relative_abundance,na.rm = TRUE))%>%
  ungroup()%>%
  mutate(Otu_genus=str_replace(Otu_genus,"_unclassified",""),
         Otu_genus=str_replace(Otu_genus,"Bacteria","Unclassified"),
         Otu_genus=str_replace(Otu_genus,"-","\n"),
         Otu_genus=factor(Otu_genus))%>%
  filter(mean_abundance>4.5)%>%
  ggplot(aes(x = fct_reorder(Otu_genus, mean_abundance, .desc = TRUE),
```

```
             y = mean_abundance,
             fill = Otu_genus)) +
  geom_bar(stat = "identity") +
  labs(x = "Otu", y = "Mean relative abundance negative controls") +
  theme_bw()+
  theme(axis.text.x= element_text(angle=60, hjust=1, size=7),
        legend.position="none",
        panel.grid = element_blank(),
        aspect.ratio = 1,
        panel.border = element_blank(),
        axis.line = element_line())+
  labs(x=NULL)+
    scale_fill_manual(values = c("#e31a1c", "#1f78b4", "#a6cee3",
                                 "#b2df8a", "#33a02c", "#fb9a99",
                                 "#fdbf6f", "#ff7f00",
                                 "#cab2d6", "#6a3d9a"))

contaminant_abundance
```

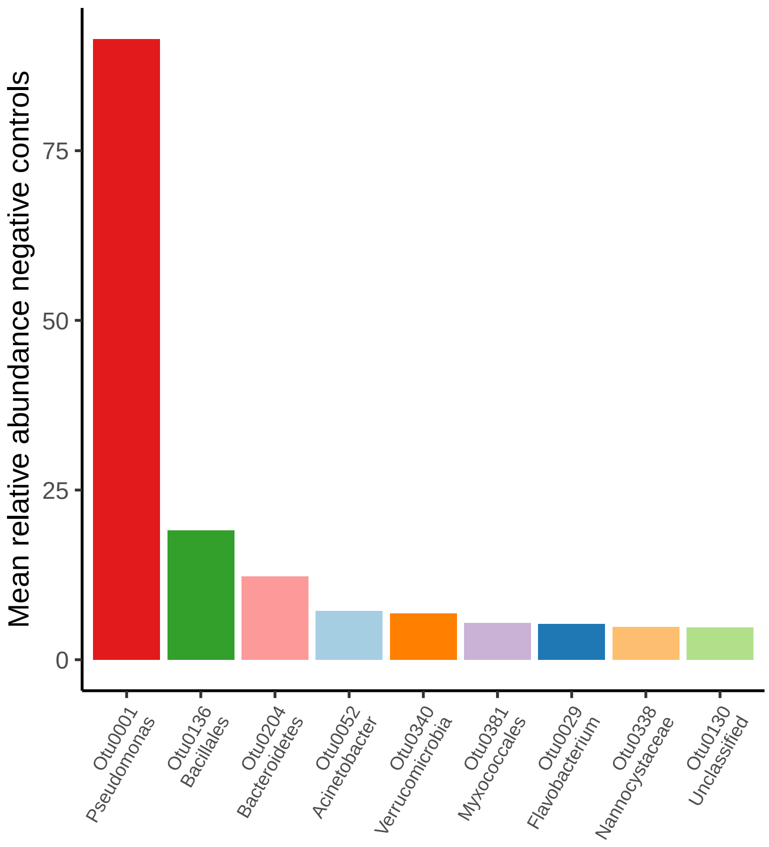


```
pseudo_v_abundance<-all_data%>%
  mutate(experiment=case_when(experiment =="AE" ~ "Elution buffer",
                              experiment =="IsoCtrl"~"Isolation control",
                              experiment == "Swab" ~ "Rectal swab",
                              experiment == "Water"~"Water"),
         experiment=factor(experiment,
```

```
                              levels=c("Water","Elution buffer",
                                       "Isolation control","Rectal swab")))%>%
  filter(Sample_ID %in% samples_for_first_plot,
         experiment != "Elution buffer")%>%
  ggplot(aes(x=ddPCR_reads_per_sample, y=Otu0001))+
  geom_point(aes(color=experiment))+
  scale_x_log10()+
  theme_bw()+
  theme(panel.grid = element_blank(),
        panel.border = element_blank(),
        axis.line = element_line(),
        legend.position = "bottom",
        axis.title.y = element_text(color="#e31a1c"),
        legend.text = element_text(size=7.5),
        aspect.ratio = 1)+
  labs(color=NULL, x="ddPCR 16S copies per sample", y ="Relative abundance Otu0001:Pseudomonas")+
  scale_color_manual(values=rev(c("#654321","#33a02c","#a6cee3")))+
  stat_cor(method="spearman",show.legend = FALSE,label.y=62.5,label.x=4.5,size=4)

joined_contamination<-ggarrange(contaminant_abundance, pseudo_v_abundance, nrow=1,
          widths=c(0.9,1),align="v")

joined_contamination
```

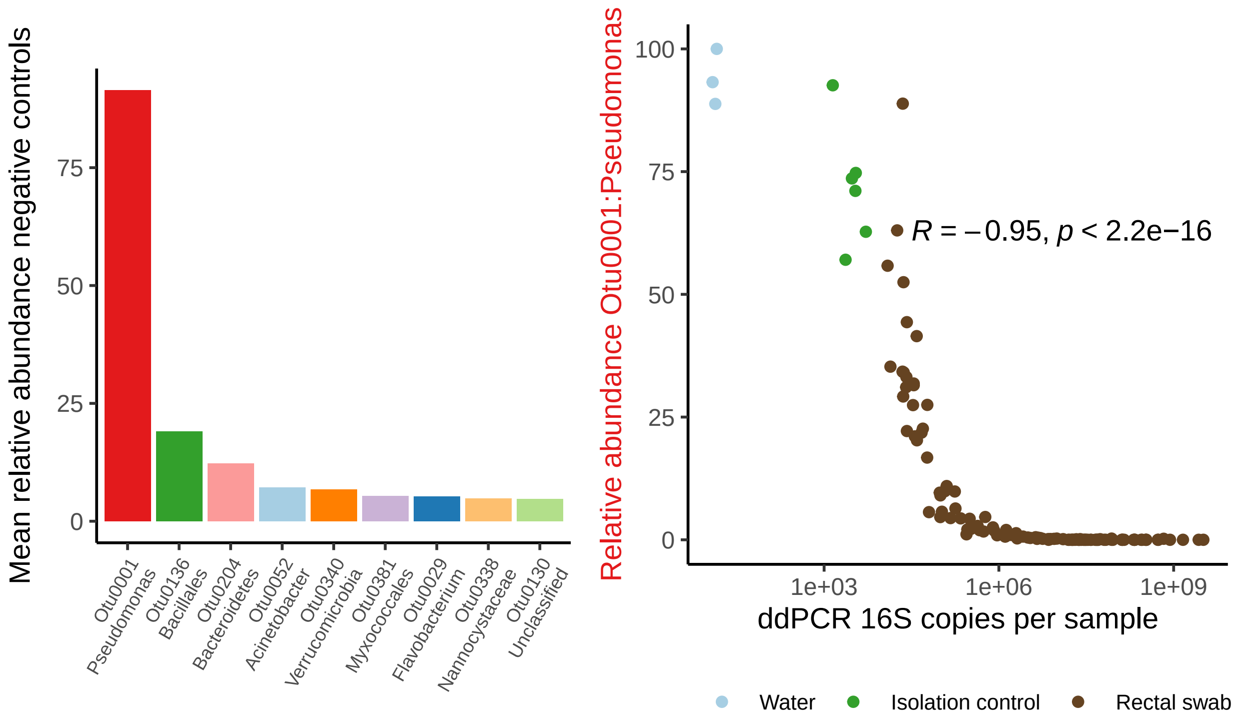

```r
#
# ggexport(joined_contamination, filename="joined_contamination_edited.pdf")
```

```r
just_initial<-all_data_swabs%>%
  filter(swab_type=="initial")

otu.swab.hel<- just_initial %>%
  dplyr::select(contains("Otu"))%>%
  decostand("hellinger")


tidy_swab_pca <- prcomp(otu.swab.hel)

loadings<-data.frame(tidy_swab_pca$rotation)%>%
  mutate(Sample_ID = rownames(.))%>%
  dplyr::select(Sample_ID,PC1, PC2)%>%
  mutate(size=sqrt(PC1^2+PC2^2))%>%
  arrange(desc(size))%>%
  head(5)%>%
  mutate(above_threshold="loading")



plot_by_burden<-data.frame(tidy_swab_pca$x)%>%
  mutate(Sample_ID = just_initial$Sample_ID)%>%
  dplyr::select(Sample_ID, PC1, PC2)%>%
  mutate(size=sqrt(PC1^2+PC2^2))%>%
  inner_join(all_data_swabs%>%dplyr::select(Sample_ID, above_threshold))%>%
  mutate(above_threshold = case_when(above_threshold == "above"~"High biomass",
                                     above_threshold == "below" ~"Low biomass"))
```

```
## Joining, by = "Sample_ID"
```

```r
find_centroids<-plot_by_burden%>%
  group_by(above_threshold)%>%
  summarize(PC1_mean = mean(PC1),
            PC2_mean = mean(PC2))


principal_component_plot<-
  ggplot(data=plot_by_burden)+
  geom_point(aes(x=PC1, y=PC2,color=above_threshold), alpha=0.5)+
  geom_point(data = find_centroids, aes(x=PC1_mean, y =PC2_mean, color=above_threshold),size=7,show.lege
  stat_ellipse(aes(x=PC1, y=PC2,color=above_threshold),type = "norm", linetype = 2, show.legend = FALSE
  theme_bw()+
  theme(panel.grid=element_blank(),
        legend.title = element_blank(),
        axis.title = element_text(size=9),
        legend.position = c(0.9,0.15))+
  scale_color_manual(values=c("#1f78b4","#e31a1c"))+
  labs(x="PC1 (11.8%) explained", y="PC2 (8.6%) explained")
```

```r
myControl <- trainControl(
  method="cv",
  number=10,
  verboseIter = TRUE
)
gridsearch_burden<-data.frame(
  mtry=seq(1:10),
  splitrule="gini",
  min.node.size=5
)

set.seed(4763)
otu_burden_model <- train(
  above_threshold~.,
  method = "ranger",
  importance="permutation",
  oob.error = TRUE,
  seed = 4763,
  trControl = myControl,
  tuneGrid = gridsearch_burden,
  data = all_data_swabs%>%
  dplyr::select(- nearZeroVar(.),-Sample_ID,
                -ddPCR_reads_per_sample, -swab_type,
                -swab_label)%>%
  mutate(pair_ID=factor(pair_ID),
         case_or_control=factor(case_or_control),
         above_threshold=factor(above_threshold))

)
```

```
## + Fold01: mtry= 1, splitrule=gini, min.node.size=5
## - Fold01: mtry= 1, splitrule=gini, min.node.size=5
## + Fold01: mtry= 2, splitrule=gini, min.node.size=5
## - Fold01: mtry= 2, splitrule=gini, min.node.size=5
## + Fold01: mtry= 3, splitrule=gini, min.node.size=5
## - Fold01: mtry= 3, splitrule=gini, min.node.size=5
## + Fold01: mtry= 4, splitrule=gini, min.node.size=5
## - Fold01: mtry= 4, splitrule=gini, min.node.size=5
## + Fold01: mtry= 5, splitrule=gini, min.node.size=5
## - Fold01: mtry= 5, splitrule=gini, min.node.size=5
## + Fold01: mtry= 6, splitrule=gini, min.node.size=5
## - Fold01: mtry= 6, splitrule=gini, min.node.size=5
## + Fold01: mtry= 7, splitrule=gini, min.node.size=5
## - Fold01: mtry= 7, splitrule=gini, min.node.size=5
## + Fold01: mtry= 8, splitrule=gini, min.node.size=5
## - Fold01: mtry= 8, splitrule=gini, min.node.size=5
## + Fold01: mtry= 9, splitrule=gini, min.node.size=5
## - Fold01: mtry= 9, splitrule=gini, min.node.size=5
## + Fold01: mtry=10, splitrule=gini, min.node.size=5
## - Fold01: mtry=10, splitrule=gini, min.node.size=5
## + Fold02: mtry= 1, splitrule=gini, min.node.size=5
## - Fold02: mtry= 1, splitrule=gini, min.node.size=5
## + Fold02: mtry= 2, splitrule=gini, min.node.size=5
```

```
## - Fold02: mtry= 2, splitrule=gini, min.node.size=5
## + Fold02: mtry= 3, splitrule=gini, min.node.size=5
## - Fold02: mtry= 3, splitrule=gini, min.node.size=5
## + Fold02: mtry= 4, splitrule=gini, min.node.size=5
## - Fold02: mtry= 4, splitrule=gini, min.node.size=5
## + Fold02: mtry= 5, splitrule=gini, min.node.size=5
## - Fold02: mtry= 5, splitrule=gini, min.node.size=5
## + Fold02: mtry= 6, splitrule=gini, min.node.size=5
## - Fold02: mtry= 6, splitrule=gini, min.node.size=5
## + Fold02: mtry= 7, splitrule=gini, min.node.size=5
## - Fold02: mtry= 7, splitrule=gini, min.node.size=5
## + Fold02: mtry= 8, splitrule=gini, min.node.size=5
## - Fold02: mtry= 8, splitrule=gini, min.node.size=5
## + Fold02: mtry= 9, splitrule=gini, min.node.size=5
## - Fold02: mtry= 9, splitrule=gini, min.node.size=5
## + Fold02: mtry=10, splitrule=gini, min.node.size=5
## - Fold02: mtry=10, splitrule=gini, min.node.size=5
## + Fold03: mtry= 1, splitrule=gini, min.node.size=5
## - Fold03: mtry= 1, splitrule=gini, min.node.size=5
## + Fold03: mtry= 2, splitrule=gini, min.node.size=5
## - Fold03: mtry= 2, splitrule=gini, min.node.size=5
## + Fold03: mtry= 3, splitrule=gini, min.node.size=5
## - Fold03: mtry= 3, splitrule=gini, min.node.size=5
## + Fold03: mtry= 4, splitrule=gini, min.node.size=5
## - Fold03: mtry= 4, splitrule=gini, min.node.size=5
## + Fold03: mtry= 5, splitrule=gini, min.node.size=5
## - Fold03: mtry= 5, splitrule=gini, min.node.size=5
## + Fold03: mtry= 6, splitrule=gini, min.node.size=5
## - Fold03: mtry= 6, splitrule=gini, min.node.size=5
## + Fold03: mtry= 7, splitrule=gini, min.node.size=5
## - Fold03: mtry= 7, splitrule=gini, min.node.size=5
## + Fold03: mtry= 8, splitrule=gini, min.node.size=5
## - Fold03: mtry= 8, splitrule=gini, min.node.size=5
## + Fold03: mtry= 9, splitrule=gini, min.node.size=5
## - Fold03: mtry= 9, splitrule=gini, min.node.size=5
## + Fold03: mtry=10, splitrule=gini, min.node.size=5
## - Fold03: mtry=10, splitrule=gini, min.node.size=5
## + Fold04: mtry= 1, splitrule=gini, min.node.size=5
## - Fold04: mtry= 1, splitrule=gini, min.node.size=5
## + Fold04: mtry= 2, splitrule=gini, min.node.size=5
## - Fold04: mtry= 2, splitrule=gini, min.node.size=5
## + Fold04: mtry= 3, splitrule=gini, min.node.size=5
## - Fold04: mtry= 3, splitrule=gini, min.node.size=5
## + Fold04: mtry= 4, splitrule=gini, min.node.size=5
## - Fold04: mtry= 4, splitrule=gini, min.node.size=5
## + Fold04: mtry= 5, splitrule=gini, min.node.size=5
## - Fold04: mtry= 5, splitrule=gini, min.node.size=5
## + Fold04: mtry= 6, splitrule=gini, min.node.size=5
## - Fold04: mtry= 6, splitrule=gini, min.node.size=5
## + Fold04: mtry= 7, splitrule=gini, min.node.size=5
## - Fold04: mtry= 7, splitrule=gini, min.node.size=5
## + Fold04: mtry= 8, splitrule=gini, min.node.size=5
## - Fold04: mtry= 8, splitrule=gini, min.node.size=5
## + Fold04: mtry= 9, splitrule=gini, min.node.size=5
```

```
## - Fold04: mtry= 9, splitrule=gini, min.node.size=5
## + Fold04: mtry=10, splitrule=gini, min.node.size=5
## - Fold04: mtry=10, splitrule=gini, min.node.size=5
## + Fold05: mtry= 1, splitrule=gini, min.node.size=5
## - Fold05: mtry= 1, splitrule=gini, min.node.size=5
## + Fold05: mtry= 2, splitrule=gini, min.node.size=5
## - Fold05: mtry= 2, splitrule=gini, min.node.size=5
## + Fold05: mtry= 3, splitrule=gini, min.node.size=5
## - Fold05: mtry= 3, splitrule=gini, min.node.size=5
## + Fold05: mtry= 4, splitrule=gini, min.node.size=5
## - Fold05: mtry= 4, splitrule=gini, min.node.size=5
## + Fold05: mtry= 5, splitrule=gini, min.node.size=5
## - Fold05: mtry= 5, splitrule=gini, min.node.size=5
## + Fold05: mtry= 6, splitrule=gini, min.node.size=5
## - Fold05: mtry= 6, splitrule=gini, min.node.size=5
## + Fold05: mtry= 7, splitrule=gini, min.node.size=5
## - Fold05: mtry= 7, splitrule=gini, min.node.size=5
## + Fold05: mtry= 8, splitrule=gini, min.node.size=5
## - Fold05: mtry= 8, splitrule=gini, min.node.size=5
## + Fold05: mtry= 9, splitrule=gini, min.node.size=5
## - Fold05: mtry= 9, splitrule=gini, min.node.size=5
## + Fold05: mtry=10, splitrule=gini, min.node.size=5
## - Fold05: mtry=10, splitrule=gini, min.node.size=5
## + Fold06: mtry= 1, splitrule=gini, min.node.size=5
## - Fold06: mtry= 1, splitrule=gini, min.node.size=5
## + Fold06: mtry= 2, splitrule=gini, min.node.size=5
## - Fold06: mtry= 2, splitrule=gini, min.node.size=5
## + Fold06: mtry= 3, splitrule=gini, min.node.size=5
## - Fold06: mtry= 3, splitrule=gini, min.node.size=5
## + Fold06: mtry= 4, splitrule=gini, min.node.size=5
## - Fold06: mtry= 4, splitrule=gini, min.node.size=5
## + Fold06: mtry= 5, splitrule=gini, min.node.size=5
## - Fold06: mtry= 5, splitrule=gini, min.node.size=5
## + Fold06: mtry= 6, splitrule=gini, min.node.size=5
## - Fold06: mtry= 6, splitrule=gini, min.node.size=5
## + Fold06: mtry= 7, splitrule=gini, min.node.size=5
## - Fold06: mtry= 7, splitrule=gini, min.node.size=5
## + Fold06: mtry= 8, splitrule=gini, min.node.size=5
## - Fold06: mtry= 8, splitrule=gini, min.node.size=5
## + Fold06: mtry= 9, splitrule=gini, min.node.size=5
## - Fold06: mtry= 9, splitrule=gini, min.node.size=5
## + Fold06: mtry=10, splitrule=gini, min.node.size=5
## - Fold06: mtry=10, splitrule=gini, min.node.size=5
## + Fold07: mtry= 1, splitrule=gini, min.node.size=5
## - Fold07: mtry= 1, splitrule=gini, min.node.size=5
## + Fold07: mtry= 2, splitrule=gini, min.node.size=5
## - Fold07: mtry= 2, splitrule=gini, min.node.size=5
## + Fold07: mtry= 3, splitrule=gini, min.node.size=5
## - Fold07: mtry= 3, splitrule=gini, min.node.size=5
## + Fold07: mtry= 4, splitrule=gini, min.node.size=5
## - Fold07: mtry= 4, splitrule=gini, min.node.size=5
## + Fold07: mtry= 5, splitrule=gini, min.node.size=5
## - Fold07: mtry= 5, splitrule=gini, min.node.size=5
## + Fold07: mtry= 6, splitrule=gini, min.node.size=5
```

```
## - Fold07: mtry= 6, splitrule=gini, min.node.size=5
## + Fold07: mtry= 7, splitrule=gini, min.node.size=5
## - Fold07: mtry= 7, splitrule=gini, min.node.size=5
## + Fold07: mtry= 8, splitrule=gini, min.node.size=5
## - Fold07: mtry= 8, splitrule=gini, min.node.size=5
## + Fold07: mtry= 9, splitrule=gini, min.node.size=5
## - Fold07: mtry= 9, splitrule=gini, min.node.size=5
## + Fold07: mtry=10, splitrule=gini, min.node.size=5
## - Fold07: mtry=10, splitrule=gini, min.node.size=5
## + Fold08: mtry= 1, splitrule=gini, min.node.size=5
## - Fold08: mtry= 1, splitrule=gini, min.node.size=5
## + Fold08: mtry= 2, splitrule=gini, min.node.size=5
## - Fold08: mtry= 2, splitrule=gini, min.node.size=5
## + Fold08: mtry= 3, splitrule=gini, min.node.size=5
## - Fold08: mtry= 3, splitrule=gini, min.node.size=5
## + Fold08: mtry= 4, splitrule=gini, min.node.size=5
## - Fold08: mtry= 4, splitrule=gini, min.node.size=5
## + Fold08: mtry= 5, splitrule=gini, min.node.size=5
## - Fold08: mtry= 5, splitrule=gini, min.node.size=5
## + Fold08: mtry= 6, splitrule=gini, min.node.size=5
## - Fold08: mtry= 6, splitrule=gini, min.node.size=5
## + Fold08: mtry= 7, splitrule=gini, min.node.size=5
## - Fold08: mtry= 7, splitrule=gini, min.node.size=5
## + Fold08: mtry= 8, splitrule=gini, min.node.size=5
## - Fold08: mtry= 8, splitrule=gini, min.node.size=5
## + Fold08: mtry= 9, splitrule=gini, min.node.size=5
## - Fold08: mtry= 9, splitrule=gini, min.node.size=5
## + Fold08: mtry=10, splitrule=gini, min.node.size=5
## - Fold08: mtry=10, splitrule=gini, min.node.size=5
## + Fold09: mtry= 1, splitrule=gini, min.node.size=5
## - Fold09: mtry= 1, splitrule=gini, min.node.size=5
## + Fold09: mtry= 2, splitrule=gini, min.node.size=5
## - Fold09: mtry= 2, splitrule=gini, min.node.size=5
## + Fold09: mtry= 3, splitrule=gini, min.node.size=5
## - Fold09: mtry= 3, splitrule=gini, min.node.size=5
## + Fold09: mtry= 4, splitrule=gini, min.node.size=5
## - Fold09: mtry= 4, splitrule=gini, min.node.size=5
## + Fold09: mtry= 5, splitrule=gini, min.node.size=5
## - Fold09: mtry= 5, splitrule=gini, min.node.size=5
## + Fold09: mtry= 6, splitrule=gini, min.node.size=5
## - Fold09: mtry= 6, splitrule=gini, min.node.size=5
## + Fold09: mtry= 7, splitrule=gini, min.node.size=5
## - Fold09: mtry= 7, splitrule=gini, min.node.size=5
## + Fold09: mtry= 8, splitrule=gini, min.node.size=5
## - Fold09: mtry= 8, splitrule=gini, min.node.size=5
## + Fold09: mtry= 9, splitrule=gini, min.node.size=5
## - Fold09: mtry= 9, splitrule=gini, min.node.size=5
## + Fold09: mtry=10, splitrule=gini, min.node.size=5
## - Fold09: mtry=10, splitrule=gini, min.node.size=5
## + Fold10: mtry= 1, splitrule=gini, min.node.size=5
## - Fold10: mtry= 1, splitrule=gini, min.node.size=5
## + Fold10: mtry= 2, splitrule=gini, min.node.size=5
## - Fold10: mtry= 2, splitrule=gini, min.node.size=5
## + Fold10: mtry= 3, splitrule=gini, min.node.size=5
```

```
## - Fold10: mtry= 3, splitrule=gini, min.node.size=5
## + Fold10: mtry= 4, splitrule=gini, min.node.size=5
## - Fold10: mtry= 4, splitrule=gini, min.node.size=5
## + Fold10: mtry= 5, splitrule=gini, min.node.size=5
## - Fold10: mtry= 5, splitrule=gini, min.node.size=5
## + Fold10: mtry= 6, splitrule=gini, min.node.size=5
## - Fold10: mtry= 6, splitrule=gini, min.node.size=5
## + Fold10: mtry= 7, splitrule=gini, min.node.size=5
## - Fold10: mtry= 7, splitrule=gini, min.node.size=5
## + Fold10: mtry= 8, splitrule=gini, min.node.size=5
## - Fold10: mtry= 8, splitrule=gini, min.node.size=5
## + Fold10: mtry= 9, splitrule=gini, min.node.size=5
## - Fold10: mtry= 9, splitrule=gini, min.node.size=5
## + Fold10: mtry=10, splitrule=gini, min.node.size=5
## - Fold10: mtry=10, splitrule=gini, min.node.size=5
## Aggregating results
## Selecting tuning parameters
## Fitting mtry = 8, splitrule = gini, min.node.size = 5 on full training set
```

```
set.seed(4763)
important_features_burden<-ranger::importance_pvalues(
   otu_burden_model$finalModel,
   method = "altmann",
   formula =  above_threshold~.,
   data = all_data_swabs%>%
  dplyr::select(- nearZeroVar(.),-Sample_ID,
                -ddPCR_reads_per_sample, -swab_type,
                -swab_label)%>%
  mutate(pair_ID=factor(pair_ID),
         case_or_control=factor(case_or_control),
         above_threshold=factor(above_threshold)))%>%
   as.data.frame()%>%
  rownames_to_column()%>%
  rename(features="rowname")
```

```
## Warning in cbind(x$variable.importance, pval): number of rows of result is not a
## multiple of vector length (arg 2)
```

```
otus_for_burden<-important_features_burden%>%
  filter(importance>0,
         pvalue<0.05)%>%
  inner_join(otu_good_taxonomy, by = c("features"="OTU"))%>%
  mutate(name=str_c(features, Genus, sep = " "))%>%
  rename(`Mean Decrease in Accuracy`="importance")%>%
  mutate(ci=`Mean Decrease in Accuracy`/qnorm(pvalue, lower.tail = FALSE),
         lower_ci=`Mean Decrease in Accuracy`-1.96*ci,
         upper_ci=`Mean Decrease in Accuracy`+1.96*ci,
         name=str_remove(name, "_unclassified"),
         name=str_remove(name, " incertae_sedis"),
         name=str_replace(name, "_"," "),
         name=factor(name),
         name=fct_reorder(name, `Mean Decrease in Accuracy`)
         )%>%
```

```
  arrange(desc(lower_ci))

important_otus<-otus_for_burden%>%
  arrange(desc(`Mean Decrease in Accuracy`))%>%
  head(15)%>%
  pull(features)

feature_importance_plot<-ggplot(otus_for_burden, aes(x=name, y = `Mean Decrease in Accuracy`))+
  geom_segment(aes(x=name, xend=name, y=lower_ci, yend=upper_ci))+
  geom_point(color="red")+
  coord_flip()+
  theme_bw()+
  theme(panel.grid=element_blank(),
        axis.text.y = element_text(size=7),
        axis.title.x = element_text(size=9))+
  labs(x=NULL,y="Mean Decrease in Accuracy")
```

```
forest_identified_otus<-just_initial%>%
  dplyr::select(Sample_ID,Otu0001,Otu0029,Otu0008,Otu0016,Otu0026,Otu0042,Otu0058,Otu0006,Otu0005,
              ddPCR_reads_per_sample)%>%
  pivot_longer(cols=contains("Otu"), names_to="OTU", values_to="rel_abund")%>%
  mutate(bacterial_type = case_when(
    OTU %in% c("Otu0001","Otu0008", "Otu0029") ~ "Common sequencing contaminant",
         OTU %in% c("Otu0016","Otu0042")~ "Common skin bacteria",
         OTU %in% c("Otu0026","Otu0058","Otu0006","Otu0005") ~"Common gut bacteria"))%>%
  inner_join(otu_good_taxonomy)%>%
  mutate(present = if_else(rel_abund >0,"Present","Absent"),
         name=str_c(OTU, Genus, sep = " "),
         name=str_remove(name,"_unclassified"),
         name=str_replace(name, "_", " "),
         name=factor(name, levels=c("Otu0001 Pseudomonas", "Otu0008 Pseudomonas",
                                    "Otu0029 Flavobacterium","Otu0016 Staphylococcus",
                                    "Otu0042 Corynebacterium","Otu0026 Lactobacillus",
                                    "Otu0058 Bacillus","Otu0006 Bacteroides","Otu0005 Akkermansia")))
```

```
## Joining, by = "OTU"
```

```
otu_burden<-forest_identified_otus%>%
  ggplot(aes(x=present, y=ddPCR_reads_per_sample))+
  geom_quasirandom(alpha=0.4,aes(color=bacterial_type))+
  facet_wrap(~name)+
  stat_compare_means(label = "p.signif", label.x = 1.5, label.y=8.9)+
  geom_boxplot(alpha=0, show.legend = FALSE,aes(color=bacterial_type))+
  scale_y_log10()+
  scale_x_discrete(labels = c("Absent","Present"))+
  theme_bw()+
  theme(panel.grid=element_blank(),
        legend.position = "bottom")+
  labs(y="16S gene copies per sample",
       x=NULL,
       color=NULL)+
  scale_color_manual(values=c("#654321","#e31a1c", "#33a02c"))
```

```
multi.plot<-ggarrange(ggarrange(principal_component_plot, feature_importance_plot),
          otu_burden,align="hv",nrow=2,heights = c(1,2))
```

```
## Warning: Graphs cannot be vertically aligned unless the axis parameter is set.
## Placing graphs unaligned.
```

```
## Warning: Graphs cannot be horizontally aligned unless the axis parameter is set.
## Placing graphs unaligned.
```

```
multi.plot
```

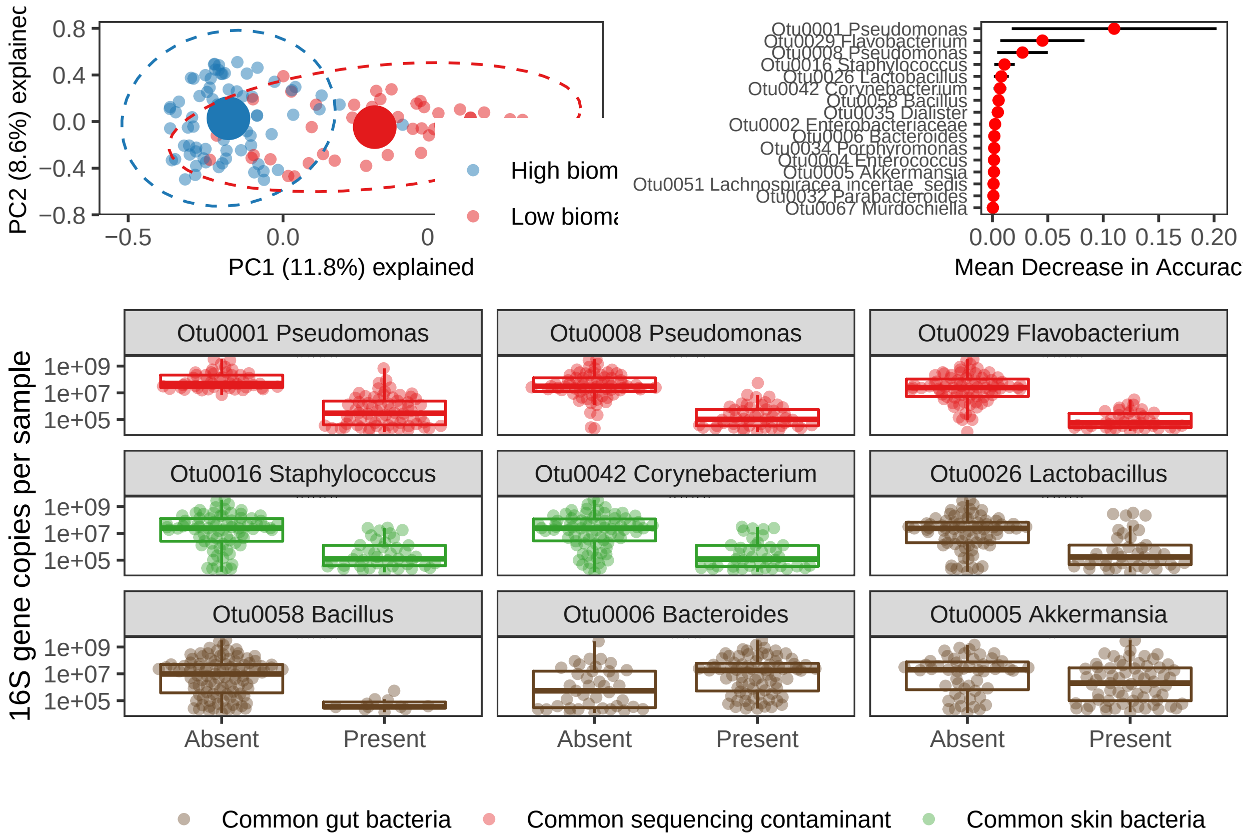


```
ggsave("features_all.pdf")
```

```
## Saving 6.5 x 4.5 in image
```

```
sofa_metadata <- read_csv("clinical_metadata_1.csv")
```

```
##
## -- Column specification --------------------------------------------------------
## cols(
##   .default = col_double(),
##   Sample_ID = col_character(),
```

```
##   culture_type = col_character(),
##   organism = col_character(),
##   above_threshold = col_character()
## )
## i Use `spec()` for the full column specifications.
```

```
cleantime <- read_csv("cleantime.csv")
```

```
##
## -- Column specification --------------------------------------------------------
## cols(
##   MRN = col_double(),
##   admit = col_character(),
##   time = col_character(),
##   Sample_ID = col_character(),
##   case_or_control = col_character(),
##   pair_ID = col_double(),
##   swab_type = col_character()
## )
```

```
sofa_metadata%>%
  dplyr::select(Augmentin:Zosyn)%>%
  pivot_longer(cols=c(Augmentin:Zosyn),names_to="abx", values_to="dose")%>%
  mutate(dose=as.numeric(dose>0))%>%
  group_by(abx)%>%
  summarize(total_doses=sum(dose))%>%
  arrange(desc(total_doses))
```

```
## # A tibble: 12 x 2
##    abx          total_doses
##    <chr>              <dbl>
##  1 Vancomycin            35
##  2 Flagyl                22
##  3 Zosyn                 20
##  4 Cefepime              18
##  5 Cefoxitin              4
##  6 Augmentin              2
##  7 Oral_Vanco             2
##  8 Meropenem              1
##  9 Doxycyline             0
## 10 Rectal_Vanco           0
## 11 Rifaximin              0
## 12 Unasyn                 0
```

```
sofa_metadata%>%
  dplyr::select(ddPCR_reads_per_sample, Augmentin:Zosyn)%>%
  pivot_longer(cols=c(Augmentin:Zosyn), names_to="antibiotic",values_to="dot")%>%
  nest(dot, ddPCR_reads_per_sample)%>%
  mutate(correlation=map(data, ~cor.test(.$dot, log(.$ddPCR_reads_per_sample))%>%
                         tidy()))%>%
  unnest(correlation)%>%
  filter(!is.na(estimate),
```

```
        antibiotic %in% c("Cefepime","Flagyl","Vancomycin","Zosyn"))%>%
  dplyr::select(antibiotic,estimate, p.value)%>%
  mutate_if(is.numeric, ~round(.,3))
```

```
## Warning: All elements of `...` must be named.
## Did you want `data = c(dot, ddPCR_reads_per_sample)`?

## Warning in cor(x, y): the standard deviation is zero

## Warning in cor(x, y): the standard deviation is zero

## Warning in cor(x, y): the standard deviation is zero

## Warning in cor(x, y): the standard deviation is zero

## # A tibble: 4 x 3
##   antibiotic estimate p.value
##   <chr>         <dbl>   <dbl>
## 1 Cefepime     -0.023   0.805
## 2 Flagyl        0.061   0.512
## 3 Vancomycin   -0.088   0.347
## 4 Zosyn        -0.257   0.005
```

```
nested<-sofa_metadata%>%
  dplyr::select(Cefepime, Flagyl, Meropenem, Vancomycin, Zosyn:rheumd, total_sofa,-MRN,-aids, -msld,-di
  mutate_if(is.character, ~as.numeric(.x))%>%
  mutate(log_burden = log(ddPCR_reads_per_sample))%>%
  dplyr::select(-ddPCR_reads_per_sample)%>%
  pivot_longer(cols=c(Cefepime:total_sofa),names_to="variable", values_to="value")%>%
  nest(-variable)%>%
  mutate(binary_data = map(data, ~mutate(.,value=if_else(value>0,1,0))))%>%
  mutate(mean_binary = map(binary_data, ~summarize(., mean=mean(value))),
         mean_continous = map(data, ~summarize(., mean=mean(value))),
         se_cont=map(data, ~summarize(.,se=sd(value)/(sqrt(n())))),
         standard_error_binary=map(binary_data, ~summarize(.,se=sd(value)/(sqrt(n())))))%>%
  unnest(mean_continous,se_cont,mean_binary, standard_error_binary)%>%
  mutate(stat = if_else(variable %in% c("age","total_sofa","charlson_score"), mean1, mean))
```

```
## Warning: All elements of `...` must be named.
## Did you want `data = c(log_burden, value)`?

## Warning: unnest() has a new interface. See ?unnest for details.
## Try `df %>% unnest(c(mean_continous, se_cont, mean_binary, standard_error_binary))`, with `mutate()`
```

```
comorbidities<-nested%>%
  filter(!(variable %in% c("Cefepime","Flagyl","Vancomycin","Zosyn","Meropenem")))%>%
  mutate(real_se = if_else(variable %in% c("age","total_sofa","charlson_score"), se, se1))%>%
  dplyr::select(variable, stat,real_se, binary_data, data)%>%
  mutate(number=if_else(variable %in% c("age","total_sofa","charlson_score"),116,stat*116),
         stat=round(stat,2),
         real_se=round(real_se,2),
```

```
        descriptor = if_else(variable %in% c("age","total_sofa","charlson_score"),real_se,number))%>%
  dplyr::select(variable, stat, descriptor, binary_data, data)

binary_comorbidities <-comorbidities%>%
  filter(!(variable %in% c("age","total_sofa","charlson_score")))

continous_comorbidities<-comorbidities%>%
  filter((variable %in% c("age","total_sofa","charlson_score")))

tested_binary_comorbidities<-binary_comorbidities%>%
  mutate(testing = map(data, ~t.test(log_burden~value, data=.)%>%
                         tidy()%>%
                         dplyr::select(p.value)))%>%
  unnest(testing)%>%
  mutate(compare_means = map(binary_data, ~group_by(.,value)%>%
                               summarize(mean_burden = mean(log_burden),
                                         se_burden = sd(log_burden)/sqrt(n()))))%>%
  unnest(compare_means)%>%
  mutate(value = if_else(value>0,"received","did_not_receive"))%>%
  mutate(mean_burden = round(mean_burden, 2),
         se_burden = round(se_burden*1.96,2))%>%
  dplyr::select(-data,-binary_data)%>%
  pivot_wider(names_from=value,values_from=c(mean_burden, se_burden))%>%
  unite("without_comorbidity",mean_burden_did_not_receive, se_burden_did_not_receive, sep="±")%>%
  unite("with_comorbidity",mean_burden_received, se_burden_received, sep="±")

tested_binary_comorbidities
```

```
## # A tibble: 20 x 6
##    variable    stat descriptor p.value without_comorbidity with_comorbidity
##    <chr>      <dbl>      <dbl>   <dbl> <chr>               <chr>
##  1 ami         0.16         18  0.0254 14.83±0.7           16.31±1.03
##  2 bmt         0.17         20  0.114  15.28±0.68          14.01±1.37
##  3 canc        0.7          81  0.835  15.17±1.22          15.02±0.71
##  4 cdiff       0.09         10  0.721  15.03±0.65          15.42±1.96
##  5 cevd        0.21         24  0.195  14.88±0.72          15.78±1.13
##  6 chf         0.33         38  0.484  14.91±0.74          15.39±1.12
##  7 copd        0.46         53  0.621  15.21±0.87          14.9±0.86
##  8 dementia    0.03          4  0.0266 14.97±0.63          17.59±1.42
##  9 diabwc      0.41         47  0.0428 14.53±0.75          15.85±1.02
## 10 GenderCode  0.45         52  0.718  15.17±0.82          14.94±0.93
## 11 hp          0.09         10  0.668  15.03±0.66          15.4±1.5
## 12 leukemia    0.26         30.  0.583  15.17±0.7           14.76±1.28
## 13 lymphoma    0.12         14  0.608  15±0.64             15.57±2.05
## 14 metacanc    0.47         54  0.0850 14.56±0.82          15.64±0.9
## 15 mld         0.1          12  0.615  15.12±0.65          14.55±2.08
## 16 pud         0.14         16  0.614  15.12±0.68          14.72±1.35
## 17 pvd         0.06          7  0.315  14.99±0.64          16.23±2.15
## 18 RaceCode    0.15         17  0.423  15.17±0.67          14.46±1.57
## 19 rend        0.4          46  0.149  14.7±0.79           15.63±0.97
## 20 rheumd      0.04          5  0.649  15.03±0.63          15.81±3.06
```

```
antibiotics<-nested%>%
  filter(variable %in% c("Cefepime","Flagyl","Vancomycin","Zosyn"))%>%
  mutate(num = map(binary_data, ~summarize(.,number = sum(value))))%>%
  unnest(num)%>%
  mutate(variable = if_else(variable == "Flagyl", "Metronidazole",variable),
         variable = if_else(variable == "Zosyn","Piperacillin-Tazobactam",variable),
         stat = round(stat, 2))%>%
  arrange(desc(number))%>%
  rename(number_received="number")%>%
  mutate(compare_means = map(binary_data, ~t.test(log_burden~value, data=.)%>%
                               tidy()%>%
                               mutate(p.value=round(p.value,digits=3))%>%
                               dplyr::select(p.value)))%>%
  unnest(compare_means)%>%
  mutate(compare_means = map(binary_data, ~group_by(.,value)%>%
                               summarize(mean_burden = mean(log_burden),
                                         se_burden = sd(log_burden)/sqrt(n()))))%>%
  unnest(compare_means)%>%
  mutate(value = if_else(value>0,"received","did_not_receive"))%>%
  mutate(mean_burden = round(mean_burden, 2),
         se_burden = round(se_burden,2))%>%
  dplyr::select(-data,-binary_data, -mean,-mean1,-se,-se1)%>%
  pivot_wider(names_from=value,values_from=c(mean_burden, se_burden))%>%
  unite("did_not_recieve_burden",mean_burden_did_not_receive, se_burden_did_not_receive, sep="±")%>%
  unite("did_receive_burden",mean_burden_received, se_burden_received, sep="±")

antibiotics
```

```
## # A tibble: 4 x 6
##   variable      stat number_received p.value did_not_recieve_b~ did_receive_bur~
##   <chr>        <dbl>           <dbl>   <dbl> <chr>              <chr>
## 1 Vancomycin    0.3               35   0.580 15.18±0.39         14.81±0.53
## 2 Metronidazo~  0.19              22   0.791 15.03±0.37         15.21±0.55
## 3 Piperacilli~  0.17              20   0.006 15.46±0.34         13.15±0.7
## 4 Cefepime      0.16              18   0.352 14.95±0.35         15.7±0.72
```

```
nested<-sofa_metadata%>%
  dplyr::select(Cefepime, Flagyl, Meropenem, Vancomycin, Zosyn:rheumd, total_sofa)%>%
  mutate_if(is.character, ~as.numeric(.x))%>%
  mutate(Sample_ID = sofa_metadata$Sample_ID)%>%
  pivot_longer(cols=-Sample_ID, names_to="variable", values_to="value")%>%
  inner_join(all_data_swabs%>%dplyr::select(Sample_ID, ddPCR_reads_per_sample))%>%
  mutate(log_reads=log(ddPCR_reads_per_sample))%>%
  dplyr::select(-Sample_ID,-ddPCR_reads_per_sample)%>%
  filter(!(variable %in% c("MRN", "Augmentin","Unasyn",
                          "Opiate_use","Doxycyline","Rifaxamin",
                          "Oral_Vanco","Rectal_Vanco","Cefoxitin")))%>%
  nest(-variable)%>%
  mutate(cor_result = map(data, ~cor.test(.$value, .$log_reads, data=.)%>%
                            tidy()))%>%
  unnest(cor_result)%>%
  dplyr::select(-statistic,-parameter,-method,-alternative)%>%
  mutate_if(is.numeric, ~round(.,3))%>%
```

```
  rename(`Pearson r`="estimate")%>%
  mutate(conf.low = as.character(conf.low),
         conf.high = as.character(conf.high),
         `(` = "(",
         `)` = ")")%>%
  unite(col="lower_ci",`(`, conf.low, sep="")%>%
  unite(col="upper_ci",conf.high,`)`,  sep="")%>%
  unite(col = "95% CI", lower_ci, upper_ci, sep="-")%>%
  filter(!is.na(`Pearson r`))
```

```
## Joining, by = "Sample_ID"

## Warning: All elements of `...` must be named.
## Did you want `data = c(value, log_reads)`?

## Warning in cor(x, y): the standard deviation is zero

## Warning in cor(x, y): the standard deviation is zero

## Warning in cor(x, y): the standard deviation is zero
```

```
nested%>%
  dplyr::select(-data)
```

```
## # A tibble: 28 x 4
##    variable   `Pearson r` p.value `95% CI`
##    <chr>            <dbl>   <dbl> <chr>
##  1 Cefepime        -0.023   0.805 (-0.205-0.16)
##  2 Flagyl           0.061   0.512 (-0.122-0.241)
##  3 Meropenem        0.042   0.652 (-0.141-0.223)
##  4 Vancomycin      -0.088   0.347 (-0.266-0.096)
##  5 Zosyn           -0.257   0.005 (-0.419--0.078)
##  6 age              0.288   0.002 (0.112-0.447)
##  7 ami              0.159   0.088 (-0.024-0.332)
##  8 bmt             -0.143   0.126 (-0.317-0.041)
##  9 canc            -0.021   0.827 (-0.202-0.162)
## 10 cdiff            0.032   0.732 (-0.151-0.213)
## # ... with 18 more rows
```

```
clinical_predictors_initial<-sofa_metadata%>%
  inner_join(cleantime%>%dplyr::select(Sample_ID,pair_ID,case_or_control))%>%
  mutate(pip_tazo= as.numeric(Zosyn>0))%>%
  dplyr::select(ddPCR_reads_per_sample, age, charlson_score,pip_tazo,pair_ID,case_or_control)%>%
  # rename(Age="age",
  #         `Charlson comorbidity score` = "charlson_score")%>%
  group_by(pair_ID)%>%
  summarize(age_diff=mean(age),
            charlson_diff=mean(charlson_score),
            burden_diff= mean(log(ddPCR_reads_per_sample)),
            pip_tazo = mean(pip_tazo>0))%>%
  mutate(pip_tazo = if_else(pip_tazo>0,"Received treatment","Did not receive treatment"))%>%
```

```
  rename(`Mean age\nmatched pair`="age_diff",
         `Mean Charlson comorbidity index\nmatched pair`="charlson_diff")%>%
  pivot_longer(cols=-c(pair_ID,pip_tazo,burden_diff),names_to="vars",values_to="values")%>%
  ggplot(aes(x=values,y=burden_diff,color=factor(pip_tazo)))+
  geom_point()+
  facet_wrap(~vars,scales="free_x",ncol=3,strip.position = "bottom")+
  theme_bw()+
  theme(panel.grid = element_blank(),
        panel.background = element_blank(),
        axis.line = element_line(),
        legend.position = "bottom",
        aspect.ratio = 1,strip.placement = "outside",
        strip.background = element_blank(),
        axis.title.y=element_text(angle=0,vjust=0.5))+
  labs(x=NULL, y="Mean 16S gene\ncopies/sample\nmatched pair\n(log scale)",color="Piperacillin-tazobact
  geom_smooth(se=FALSE,method="lm",show.legend = FALSE)+
  scale_color_manual(values = c("#1f78b4","#e31a1c","#000000"))
```

```
## Joining, by = "Sample_ID"
```

```
clinical_predictors_initial
```

```
## `geom_smooth()` using formula 'y ~ x'
```

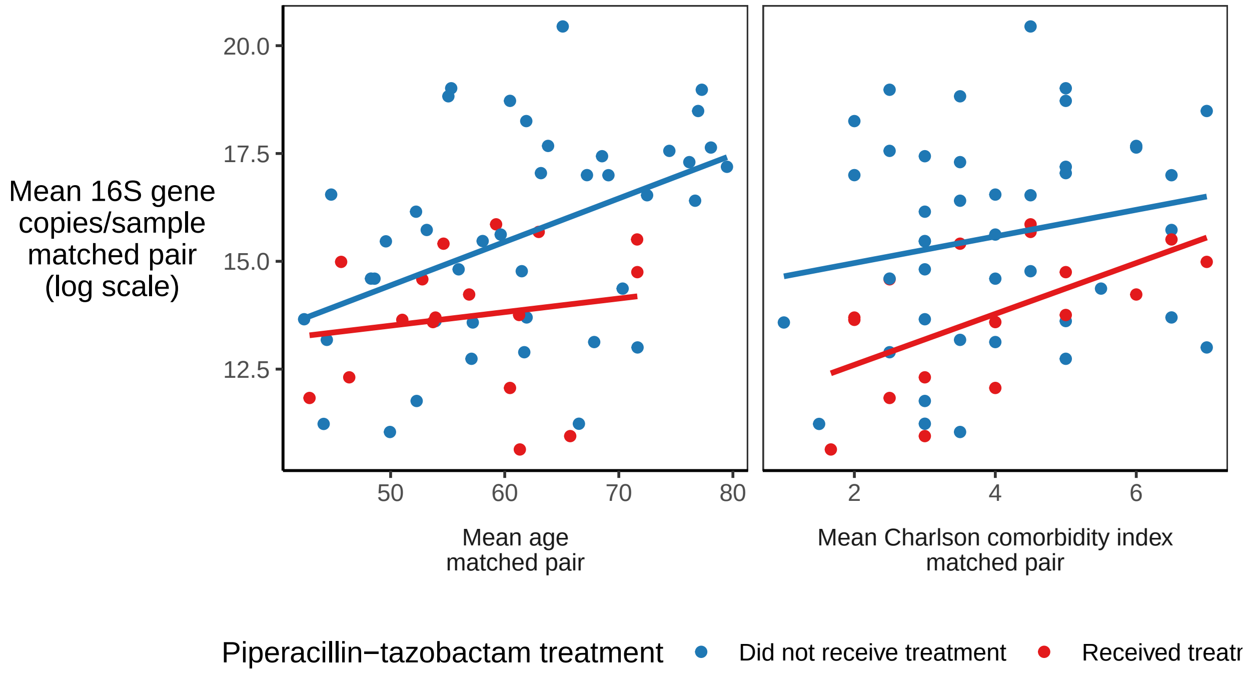

```
# ggsave("figure_3_mixed.pdf")
```

```
kaplan_meier_fit <- survfit(Surv(survival_time, infection) ~ t, data = sofa_metadata%>%
                              # filter(survival_time<=25)%>%
                              rename(t="above_threshold"))
survp<-ggsurvplot(kaplan_meier_fit,conf.int = FALSE,          # Add confidence interval
  pval = TRUE,
  risk.table = T,
  xlim=c(0,21),
  tables.y.text = FALSE,
  break.time.by = 7,
  censor = F,
  legend = "none",
  palette = c("#e31a1c","#1f78b4"),
  ggtheme = theme_bw()+theme(panel.grid = element_blank()))

survp
```

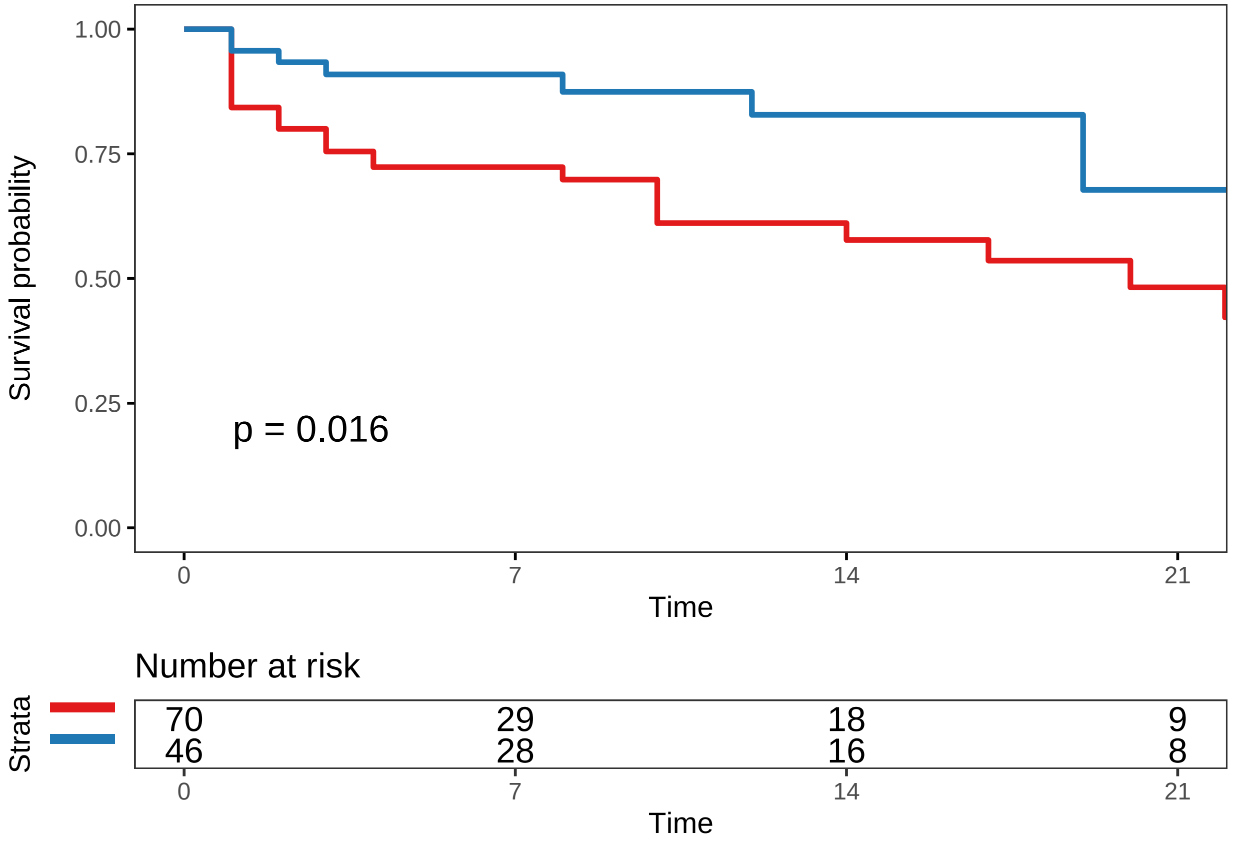


```
cleantime<-read_csv("cleantime.csv")
```

```
##
## -- Column specification --------------------------------------------------------
## cols(
##   MRN = col_double(),
```

```
##   admit = col_character(),
##   time = col_character(),
##   Sample_ID = col_character(),
##   case_or_control = col_character(),
##   pair_ID = col_double(),
##   swab_type = col_character()
## )
```

```
Single_Patient_Summary <- read_csv("VRE-Single-Patient-Summary.csv")%>%
  inner_join(cleantime%>%
               dplyr::select(Sample_ID, time)%>%
               mutate(time = mdy_hm(time)),
             by = c("swab_1_time"="time"))%>%
  inner_join(sofa_metadata)%>%
  inner_join(all_data_swabs%>%dplyr::select(Sample_ID, case_or_control,pair_ID))
```

```
##
## -- Column specification --------------------------------------------------------
## cols(
##   hosp_id = col_double(),
##   APS_Score = col_double(),
##   Comorbidity_Score = col_double(),
##   APACHE_Score = col_double(),
##   days_between_swabs = col_double(),
##   swab_1_time = col_datetime(format = ""),
##   swab_2_time = col_datetime(format = ""),
##   spo2_count = col_double(),
##   pao2_count = col_double(),
##   fio2_count = col_double(),
##   pao2_count_24 = col_double(),
##   invasive = col_double(),
##   supl = col_double(),
##   ra = col_double(),
##   hfnc = col_double(),
##   noninvasive = col_double()
## )
```

```
## Joining, by = "Sample_ID"
## Joining, by = "Sample_ID"
```

```
reason_for_admit <- read_csv("reason_for_admit.csv")%>%
  inner_join(cleantime%>%
               dplyr::select(MRN, Sample_ID))%>%
  dplyr::select(-MRN)
```

```
##
## -- Column specification --------------------------------------------------------
## cols(
##   MRN = col_double(),
##   ProblemDescription = col_character(),
##   Category = col_character()
## )
```

```
## Joining, by = "MRN"
```

```
admit_dx<-
  inner_join(Single_Patient_Summary, reason_for_admit)%>%
  distinct()%>%
  mutate(Category = as.character(Category))%>%
  mutate(reason_for_admit =case_when(
           Category == "connective_tissue"~"baseline",
           Category %in% c("cardiac","respiratory_failure","dehydration")~
             "cardio_pulm",
           Category %in% c("gi_bleed","GI_anatomic")~"gastro",
           Category %in% c("pain","lymphoma","solid_malignancy",
                           "acute_leukemia")~"malignancy",
           Category =="transplant"~"transplant",
           Category =="neurologic"~"neurologic",
           Category =="trauma"~"trauma",
           str_detect(Category, "infection")~"sepsis",
         ),
         reason_for_admit=factor(reason_for_admit),
         reason_for_admit=fct_reorder(reason_for_admit, ddPCR_reads_per_sample),
         cardio_pulm = if_else(reason_for_admit=="cardio_pulm",1,0),
         gastro=if_else(reason_for_admit =="gastro",1,0),
         malignancy=if_else(reason_for_admit=="malignancy",1,0),
         trauma=if_else(reason_for_admit=="trauma",1,0),
         sepsis =if_else(str_detect(reason_for_admit, "sepsis"),1,0),
         neuro = if_else(reason_for_admit=="neurologic",1,0),
         transplant=if_else(reason_for_admit=="transplant",1,0))
```

```
## Joining, by = "Sample_ID"
```

```
survival<-coxph(Surv(survival_time, infection) ~ log(ddPCR_reads_per_sample)+
        total_sofa+Comorbidity_Score+case_or_control+
          +Zosyn+sepsis+
          frailty(factor(pair_ID), distribution = "gaussian",
                  sparse = FALSE, method = "reml"),
      data = admit_dx)


conf_int_survival<-survival%>%
  confint()%>%
  data.frame()%>%
  mutate(across(is.numeric, ~exp(.)))%>%
  rownames_to_column(var="variable")%>%
  filter(!str_detect(variable,"gauss"))%>%
  rename(lower_ci="X2.5..",
         upper_ci="X97.5..",
         )%>%
  mutate(range = (upper_ci-lower_ci)/2)
```

```
## Warning: Predicate functions must be wrapped in `where()`.
##
##   # Bad
##   data %>% select(is.numeric)
```

```
##
##   # Good
##   data %>% select(where(is.numeric))
##
## i Please update your code.
## This message is displayed once per session.
```

```
coef_survival<-survival%>%
  summary()%>%
  coef()%>%
  data.frame()%>%
  rownames_to_column(var="variable")%>%
  mutate(variable = if_else(variable=="log(ddPCR_reads_per_sampl","log(ddPCR_reads_per_sample)",variable
  mutate(coef=exp(coef),
         se.coef.=exp(se.coef.),
         se2=exp(se2))



mixed_effects_survival<-inner_join(coef_survival, conf_int_survival, by ="variable")%>%
  mutate(variable = str_replace(variable, "case_or_controlcase","vre_colonized"))%>%
  dplyr::select(variable, coef, p, lower_ci, upper_ci, range)%>%
  mutate(across(where(is.numeric), ~round(.,3)))%>%
  mutate(lower_ci = as.character(lower_ci),
         upper_ci = as.character(upper_ci))%>%
  mutate(left_parenth="(",
         right_parenth=")")%>%
  unite(left_side, c("left_parenth", "lower_ci"),sep="")%>%
  unite(right_side, c("upper_ci", "right_parenth"),sep="")%>%
  unite(ninety_five_conf, c("left_side","right_side"),sep="-")

mixed_effects_survival
```

```
##                      variable  coef     p ninety_five_conf range
## 1 log(ddPCR_reads_per_sample) 1.213 0.003    (1.067-1.378) 0.155
## 2                  total_sofa 0.987 0.845    (0.866-1.125) 0.129
## 3           Comorbidity_Score 1.017 0.650    (0.947-1.092) 0.072
## 4               vre_colonized 0.632 0.211    (0.308-1.298) 0.495
## 5                       Zosyn 2.320 0.082    (0.899-5.986) 2.543
## 6                      sepsis 2.203 0.056    (0.981-4.944) 1.981
```

```
forest_plot<-inner_join(coef_survival, conf_int_survival, by ="variable")%>%
  mutate(variable = factor(variable),
         variable = fct_reorder(variable, coef))%>%
  mutate(variable = case_when(
    variable=="Zosyn"~"Piperacillin-\nTazobactam",
    variable=="sepsis"~"Sepsis on\nadmission",
    variable=="log(ddPCR_reads_per_sample)"~"Bacterial density\nlog(16S copies/\nsample)",
    variable=="Comorbidity_Score"~"Charlson\ncomorbidity\nindex",
    variable=="total_sofa"~"SOFA Score",
    variable=="case_or_controlcase"~"VRE colonized"
  ))%>%
  mutate(variable = factor(variable),
```

```
         variable = fct_reorder(variable, coef))%>%
  ggplot(aes(x=variable))+
  geom_point(aes(y=coef), color="#e31a1c", size=2)+
  geom_segment(aes(y=lower_ci, yend=upper_ci, x=variable, xend=variable))+
  theme_bw()+
  theme(panel.grid=element_blank(),
        panel.border = element_blank(),
        axis.ticks.y = element_blank(),
        axis.line.x = element_line(),
        aspect.ratio = 1)+
  coord_flip()+
  geom_hline(aes(yintercept=1), linetype="dashed")+
  labs(y="Hazard Ratio",
       x=NULL)

forest_plot
```

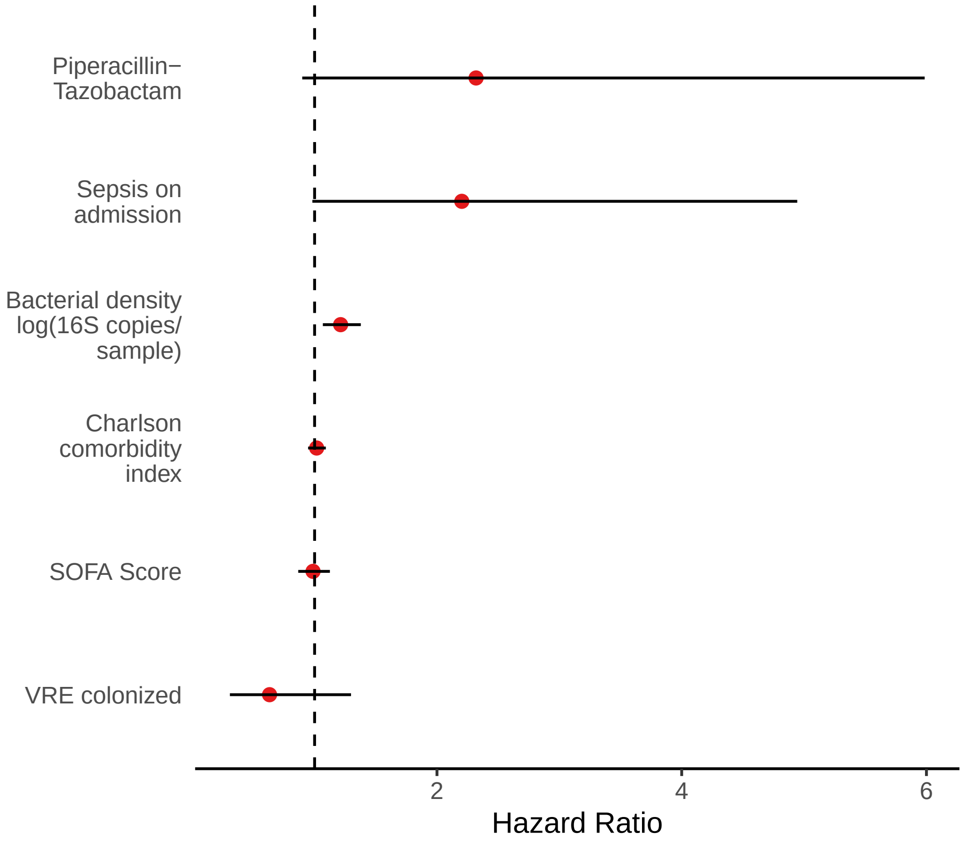


```
lmer_model<-lmerTest::lmer(log(ddPCR_reads_per_sample) ~
                 (1|pair_ID)+total_sofa+Zosyn+
                   case_or_control+age+cardio_pulm+
                 +neuro+trauma+charlson_score+
                 sepsis+transplant+gastro+
                 malignancy,
               data = admit_dx%>%
                 mutate(pair_ID=factor(pair_ID)))
```

```
## Registered S3 methods overwritten by 'lme4':
##   method                          from
##   cooks.distance.influence.merMod car
##   influence.merMod                car
##   dfbeta.influence.merMod         car
##   dfbetas.influence.merMod        car
```

```
summary(lmer_model)
```

```
## Linear mixed model fit by REML. t-tests use Satterthwaite's method [
## lmerModLmerTest]
## Formula: log(ddPCR_reads_per_sample) ~ (1 | pair_ID) + total_sofa + Zosyn +
##     case_or_control + age + cardio_pulm + +neuro + trauma + charlson_score +
##     sepsis + transplant + gastro + malignancy
##    Data: admit_dx %>% mutate(pair_ID = factor(pair_ID))
##
## REML criterion at convergence: 582.1
##
## Scaled residuals:
##     Min      1Q  Median      3Q     Max
## -1.9731 -0.6483  0.1191  0.5972  2.0130
##
## Random effects:
##  Groups   Name        Variance Std.Dev.
##  pair_ID  (Intercept) 0.2783   0.5275
##  Residual             8.9892   2.9982
## Number of obs: 119, groups:  pair_ID, 59
##
## Fixed effects:
##                      Estimate Std. Error        df t value Pr(>|t|)
## (Intercept)           9.44783    3.33395 105.84727   2.834  0.00551 **
## total_sofa            0.07026    0.10712 101.21330   0.656  0.51339
## Zosyn                -1.84047    0.76022 102.08869  -2.421  0.01725 *
## case_or_controlcase  -0.07260    0.59477  74.33851  -0.122  0.90318
## age                   0.04270    0.02084 105.03598   2.049  0.04294 *
## cardio_pulm           0.67448    3.23880 105.74564   0.208  0.83543
## neuro                 2.40806    3.68542 105.92980   0.653  0.51491
## trauma                1.35297    3.46025 105.79873   0.391  0.69658
## charlson_score        0.45054    0.15258  95.62554   2.953  0.00396 **
## sepsis                1.44459    3.21480 105.69914   0.449  0.65409
## transplant           -0.52552    3.62682 105.99196  -0.145  0.88507
## gastro                2.30090    3.29014 102.87953   0.699  0.48592
## malignancy            0.40270    3.19721 105.54270   0.126  0.90001
## ---
## Signif. codes:  0 '***' 0.001 '**' 0.01 '*' 0.05 '.' 0.1 ' ' 1
```

```
##
## Correlation matrix not shown by default, as p = 13 > 12.
## Use print(x, correlation=TRUE)  or
##     vcov(x)        if you need it
```

```r
plot(lmer_model)
```

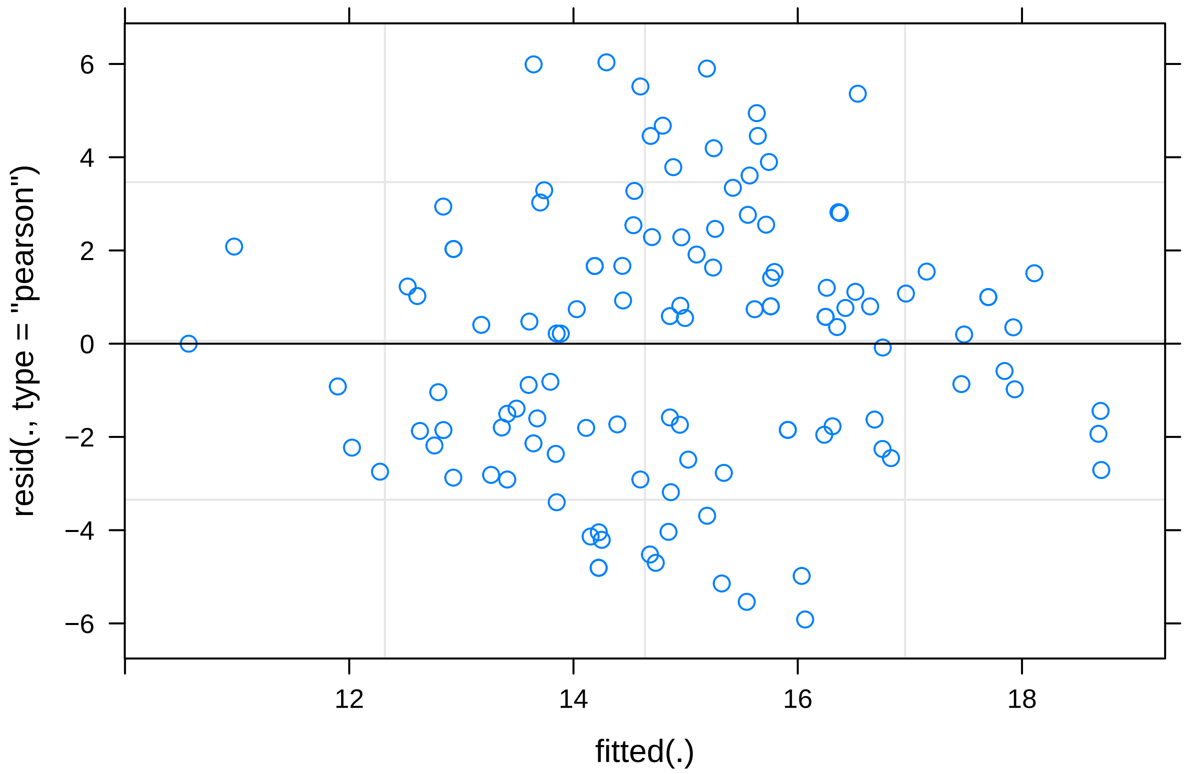


```r
coefficients<-summary(lmer_model)%>%
  coef()%>%
  data.frame()%>%
  rownames_to_column(var="variable")%>%
  rename(p_value="Pr...t..")


confidence_interval<-confint(lmer_model)%>%
               data.frame()%>%
              rownames_to_column(var="variable")%>%
  rename(lower_ci="X2.5..",
         upper_ci="X97.5..",
         )%>%
  mutate(range = (upper_ci-lower_ci)/2)
```

```
## Computing profile confidence intervals ...
```

```r
mixed_effects<-inner_join(coefficients, confidence_interval, by ="variable")%>%
  mutate(variable=str_remove(variable, "reason_for_admit"),
         variable = str_replace(variable, "case_or_controlcase","vre_colonized"))%>%
  dplyr::select(variable, Estimate, p_value, lower_ci, upper_ci, range)%>%
  mutate(across(where(is.numeric), ~round(.,3)))%>%
```

```
  mutate(lower_ci = as.character(lower_ci),
         upper_ci = as.character(upper_ci))%>%
  mutate(left_parenth="(",
         right_parenth=")")%>%
  unite(left_side, c("left_parenth", "lower_ci"),sep="")%>%
  unite(right_side, c("upper_ci", "right_parenth"),sep="")%>%
  unite(ninety_five_conf, c("left_side","right_side"),sep="-")

mixed_effects
```

```
##           variable Estimate p_value ninety_five_conf range
## 1      (Intercept)    9.448   0.006    (3.241-15.69) 6.224
## 2       total_sofa    0.070   0.513    (-0.129-0.27) 0.200
## 3            Zosyn   -1.840   0.017  (-3.266--0.423) 1.421
## 4     vre_colonized   -0.073   0.903   (-1.183-1.056) 1.120
## 5              age    0.043   0.043    (0.004-0.083) 0.040
## 6      cardio_pulm    0.674   0.835   (-5.421-6.707) 6.064
## 7            neuro    2.408   0.515   (-4.534-9.273) 6.903
## 8           trauma    1.353   0.697   (-5.104-7.798) 6.451
## 9   charlson_score    0.451   0.004     (0.161-0.74) 0.290
## 10          sepsis    1.445   0.654   (-4.582-7.428) 6.005
## 11      transplant   -0.526   0.885   (-7.275-6.276) 6.776
## 12          gastro    2.301   0.486   (-3.944-8.433) 6.188
## 13      malignancy    0.403   0.900   (-5.607-6.357) 5.982
```

```
microbiome<-sofa_metadata%>%
  dplyr::select(Sample_ID, infection, culture_type,
                organism, Opiate_use:mld, above_threshold)%>%
  dplyr::select(-age)%>%
  mutate(across(where(is.numeric),~if_else(.>0,1,0)))%>%
  inner_join(all_data_swabs%>%
               dplyr::select(Sample_ID,
                             contains("Otu"),
                             ddPCR_reads_per_sample))%>%
  # dplyr::select(-Sample_ID)%>%
  mutate(contaminant = if_else(Otu0001>0,1,0),
         above_threshold= if_else(ddPCR_reads_per_sample>10^6,1,0))%>%
  inner_join(all_data_swabs%>%
               dplyr::select(pair_ID, Sample_ID))
```

```
## Joining, by = "Sample_ID"
## Joining, by = "Sample_ID"
```

```
micro.hel<-microbiome%>%
  dplyr::select(contains("Otu"))%>%
  decostand(.,method="hellinger")



micro<-microbiome%>%
  dplyr::select(-contains("Otu"))
```

```r
permanova<-adonis(micro.hel~infection,strata=micro$pair_ID,
                  data=micro,by="terms",permutations = 9999)


head<-coefficients(permanova)%>%
  as.data.frame()%>%
  rownames_to_column("variable")%>%
  filter(variable == "infection")%>%
  pivot_longer(cols=contains("Otu"),names_to="Otu",
               values_to = "importance")%>%
  arrange(importance)%>%
  inner_join(otu_genus_link)%>%
  head()
```

```
## Joining, by = "Otu"
```

```r
tail<-coefficients(permanova)%>%
  as.data.frame()%>%
  rownames_to_column("variable")%>%
  filter(variable == "infection")%>%
  pivot_longer(cols=contains("Otu"),names_to="Otu",
               values_to = "importance")%>%
  arrange(importance)%>%
  inner_join(otu_genus_link)%>%
  tail()
```

```
## Joining, by = "Otu"
```

```r
rbind(head, tail)%>%
  mutate(protective_or_not=if_else(importance>0, "increased in infection",
                                   "decreased in infection"))%>%
  mutate(Otu_genus=factor(Otu_genus))%>%
  mutate(Otu_genus = fct_reorder(Otu_genus,importance))%>%
  ggplot(aes(x=Otu_genus, y = importance, fill=factor(protective_or_not)))+
  geom_col()+
  coord_flip()+
  theme_bw()+
  theme(aspect.ratio = .5,
        legend.position = "bottom")+
  labs(fill=NULL, x=NULL, y ="Importance")
```

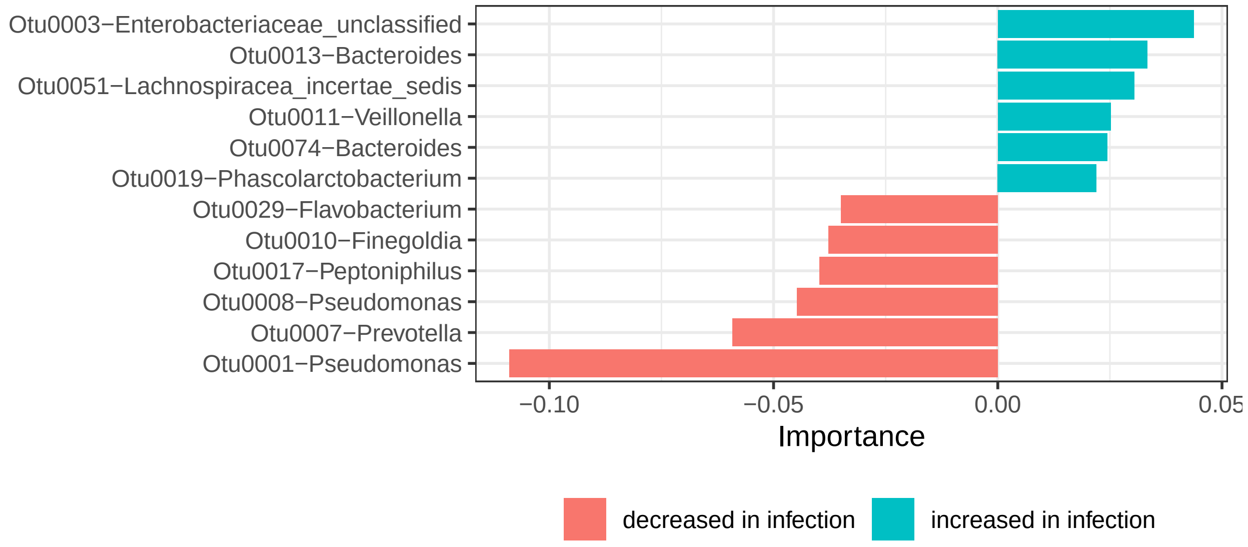


```
infections_df<-all_data_swabs%>%
  dplyr::select(Sample_ID, swab_type,Otu0002,Otu0003)%>%
  inner_join(sofa_metadata)%>%
  mutate(
    staph = as.numeric(str_detect(organism, "Staph")),
    strep = as.numeric(str_detect(organism, "Strep")),
    pseudo = as.numeric(str_detect(organism, "Pseudo")),
    enterococcus = as.numeric(str_detect(organism, "Enterococcus")),
    klebsiella = as.numeric(str_detect(organism, "Klebsiella")),
    ecoli = as.numeric(str_detect(organism, "Escherichia")),
    none = as.numeric(str_detect(organism, "none")),
    enterobacter =as.numeric(str_detect(organism, "Enterobacter"))
  )%>%
  pivot_longer(cols=contains("Otu"), names_to = "Otu",
               values_to = "relative_abundance")%>%
  filter(Otu %in% c("Otu0002","Otu0003"))%>%
  group_by(Sample_ID, Otu)%>%
  summarize(ecoli = max(ecoli),
            klebsiella=max(klebsiella),
            abundance = sum(relative_abundance))%>%
  pivot_longer(
    cols=c(ecoli, klebsiella),
    names_to="organism",
    values_to="infection")
```

```
## Joining, by = "Sample_ID"
```

```
## `summarise()` has grouped output by 'Sample_ID'. You can override using the `.groups` argument.
```

```
sig<-infections_df%>%
  mutate(infection = factor(infection))%>%
  nest(-organism,-Otu)%>%
  mutate(wilcox = map(data, ~wilcox.test(abundance~infection, data=.)%>%
                        tidy()))
```

```
## Warning: All elements of `...` must be named.
## Did you want `data = c(Sample_ID, abundance, infection)`?
```

```
infections_df%>%
  filter(Otu=="Otu0002"& organism=="ecoli"|
         Otu=="Otu0003"& organism=="klebsiella")%>%
  mutate(infection = if_else(infection==1,"Infected","Uninfected"),
         infection = factor(infection),
         organism = if_else(organism=="ecoli","OTU0002-E.coli",
                            "OTU0004-Klebsiella"))%>%
  ggplot(aes(x=infection, y = abundance, fill=factor(organism)))+
  geom_boxplot(alpha=0.25)+
  geom_point()+
  theme_bw()+
  theme(legend.position = "none",
        panel.grid = element_blank(),
        axis.title.y=element_text(angle=0, vjust=0.5),
        aspect.ratio = 1)+
  facet_wrap(~organism)+
  labs(y="Relative\nabundance\nof OTU",
       x=NULL)+
  scale_fill_manual(values=c("#1f78b4","#e31a1c"))+
  stat_compare_means()
```

```
## Warning: Unknown or uninitialised column: `p`.

## Warning: Computation failed in `stat_compare_means()`:
## argument "x" is missing, with no default

## Warning: Unknown or uninitialised column: `p`.

## Warning: Computation failed in `stat_compare_means()`:
## argument "x" is missing, with no default
```

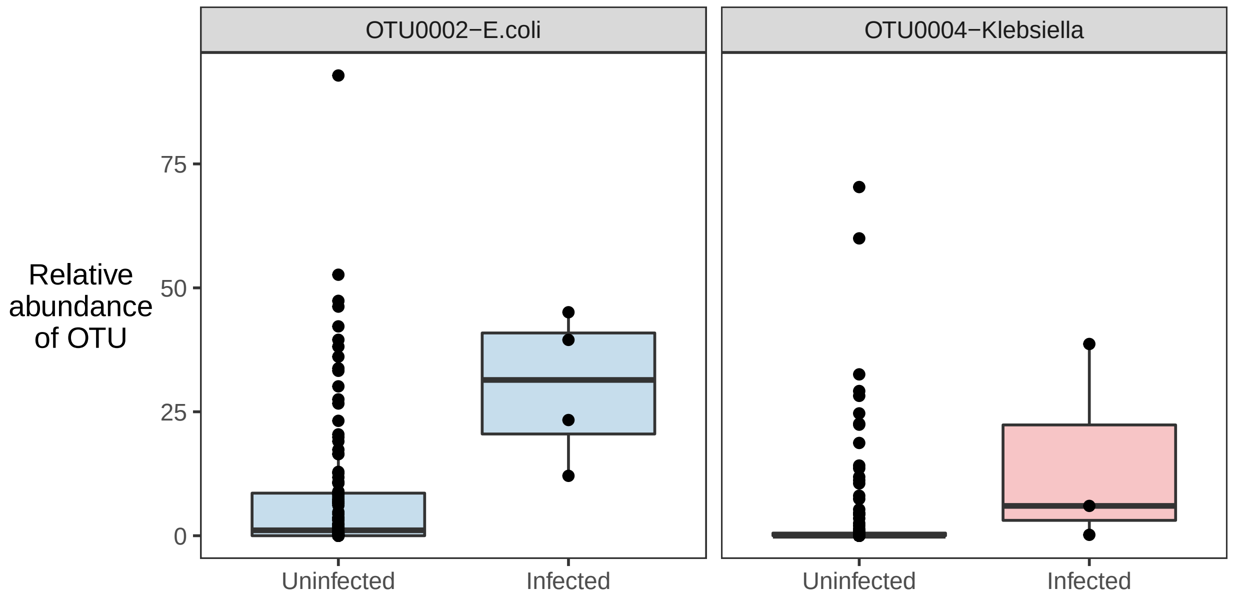


```
# ggsave("figure_7.pdf")
```
